# Beverage Consumption Patterns among U.S. Adolescents and Adults from a New 24-h Beverage Recall Survey Compared to the National Health and Nutrition Examination Survey (NHANES) 2017–2018

**DOI:** 10.3390/nu15163561

**Published:** 2023-08-12

**Authors:** Xiaoyu Bi, Benjamin J. K. Davis, Leila M. Barraj, Devanathan Srinivasan, Parvati Mahadev, Preeti Mathew, Dibyendu Mishra, Carolyn G. Scrafford, Nga L. Tran, Maia M. Jack

**Affiliations:** 1 Center for Chemical Regulation & Food Safety, Exponent, Inc., Washington, DC 20036, USAcscrafford@exponent.com (C.G.S.); ntran@exponent.com (N.L.T.); 2 Brandscapes Worldwide, Mumbai 400093, India; devanathan.srinivasan@brand-scapes.com (D.S.); parvati.mahadev@brand-scapes.com (P.M.); preeti.mathew@brand-scapes.com (P.M.); dibyendu.mishra@brand-scapes.com (D.M.); 3 Science and Regulatory Affairs, American Beverage Association, Washington, DC 20004, USA

**Keywords:** beverages, survey, NHANES, United States, teenagers, adults, dietary recall

## Abstract

Beverages are major dietary components of the United States (U.S.) population. Understanding the current consumption pattern of beverages is an important element in supporting healthy diets. Our objective was to assess the validity of the 24-h beverage consumption recall data collected in 2021 through a self-administered online questionnaire (referred to as the American Beverage Association-Brandscapes Worldwide survey, ABA-BSW) by comparing it to the 24-h dietary recall data collected in the 2017–2018 National Health and Nutrition Examination Survey (NHANES). Summary statistics on the reported consumption amounts and consumption occasions (COs) of 13 beverage types (e.g., bottled water, carbonated soft drinks (CSD), tea, and others) by participants aged 13–64 years were compared between ABA-BSW (*n* = 20,553) and NHANES (*n* = 4437). The average daily consumption amount among consumers of all 13 beverage types combined was higher in ABA-BSW than in NHANES (1903 mL/day vs. 1704 mL/day). Within each beverage type, the average daily consumption amounts among consumers were generally lower in ABA-BSW except for CSD, plant-based drinks, and still juices and fruit-flavored drinks. Compared to NHANES, ABA-BSW participants reported consuming a wider variety of beverage groups, a higher number of COs per day, and lower consumption amounts within a given CO. Overall, beverage consumption patterns observed in ABA-BSW and NHANES were generally similar, supporting the design and implementation of the former survey. Further, the ABA-BSW data provide additional information on the within-day temporal beverage consumption patterns among adolescents and adults in the U.S. Differences in the observed consumption patterns between the surveys may be the result of various factors, including the survey implementation method, a consumption pattern shift between the survey time periods, beverage type availability, and impact of the COVID-19 pandemic on dietary patterns.

## 1. Introduction

The National Health and Nutrition Examination Survey (NHANES) is conducted by the National Center for Health Statistics (NCHS) and is designed to provide information on the health and nutritional status of the United States (U.S.) population [1]. NCHS implements a complex survey design with statistical weights to ensure that nutritional and health estimates from the survey participant data are representative of the U.S. NCHS collects data on socio-demographics as well as anthropometric measures, including height and weight. The What We Eat in America (WWEIA) component of NHANES is the primary source of publicly available food, beverage, and nutrient intake data in the U.S. [1]. Since the 2003–2004 survey cycle, NCHS has recorded the amount of food and beverage consumed by an individual in a given consumption occasion (CO) via two non-consecutive 24-h dietary recalls implemented in WWEIA. NCHS has also administered a food frequency questionnaire (FFQ) that records the frequency of consumption of fish and shellfish in the previous 30 days. Between the 2003–2006 survey cycles, NCHS administered an additional FFQ to collect frequency of consumption data of several foods and beverage categories over the prior 12 months. To preserve the confidentiality of NHANES participants, the publicly released data do not include geographical identifiers. 

Consumption data from NHANES are extensively used by researchers in studies to evaluate intake patterns [2,3,4,5,6,7,8,9,10,11,12,13,14,15,16,17]. In these studies, consumption was expressed either on a short-term intake basis, (e.g., per CO, meal or time of day, or 24-h) or on a usual intake basis derived either by applying statistical models developed by the National Cancer Institute (NCI) [18] or by combining gram amounts per CO obtained from the NHANES 24-h dietary recall data with a dataset of the frequency of consumption. Since recent NHANES cycles do not include a comprehensive FFQ, frequency data from other national surveys, including the National Eating Trends survey conducted by The NPD Group [19,20,21], have been used to estimate usual intakes of foods. 

At the request of the American Beverage Association (ABA), Brandscapes Worldwide (BSW) conducted a dietary survey in 2021, henceforth referred to as the ABA-BSW survey (ABA-BSW). The survey collected data on beverage consumption in the previous 24-h as well as on the frequency of beverage consumption in the past year among a nationally representative sample of the U.S. population ages 13–64 years (y). The primary objective of the survey was to provide recent data on non-alcoholic beverage consumption patterns, including details on brands and types of beverages (e.g., ready-to-drink, sugar-sweetened beverages (SSB), regular carbonated soft drinks (CSD), low- and no-calorie sweetened carbonated soft drinks (LNCS CSD), etc.). The survey was designed to allow for the estimation of beverage consumption amounts among the U.S. population (13–64 y) as well as within U.S. geographic regions. The data collected by the survey can be used to estimate the consumption of individual beverage types (e.g., soft drinks or juices, sparkling water, etc.) and beverage constituents (e.g., ingredients, nutrients). 

The purpose of the current study was to assess similarities and differences between the 24-h beverage consumption recall data collected in ABA-BSW and the 24-h beverage consumption recall data from NHANES 2017–2018, the most recent U.S. dietary survey at the time of this analysis. We expected that the comparison would affirm the validity of the ABA-BSW survey data by showing overall similar consumption patterns from a well-established continuous dietary survey.

## 2. Materials and Methods

### 2.1. ABA-BSW

The ABA-BSW was conducted online during a 9-week period from 17 September 2021 to 23 November 2021. The data were collected through a self-administered questionnaire sent to volunteers from BSW’s panel pool of ~40 million non-institutionalized people in the U.S. The questionnaire used an interactive design to maximize respondent engagement, aid accurate recall, and minimize the likelihood of incomplete responses. A quota design was used to include a representative number of participants from all U.S. states based on demographic characteristics, including sex, race, age, and income groups. Those eligible to participate in the online survey included all individuals 13–64 y except those that worked at an advertising or market research agency or beverage company. The survey sample was restricted to this age range primarily because children under the age of 13 y require parental consent. Further, participants 65 y and older were excluded due to concerns that significant portions of this age group could have difficulty navigating the interface. Note that this concern is not relevant for NHANES as its dietary assessment is guided by a trained interviewer. The ABA-BSW survey was administered in such a fashion as to ensure adequate data coverage across all days of the week. 

The structure of the survey and the information collected are explained briefly below. A more in-depth overview of the design and interface of the survey is provided in Supplemental File S1. The survey was divided into three sections. In Section 1, data on several socio-demographic (age, sex, marital status, pregnancy status, household size, employment status, state of residence, urban/rural residential limits, race/ethnicity, income) measures and anthropometric (weight, height) measures were collected. In Section 2, survey participants were guided through a 24-h dietary recall questionnaire via a structured set of questions. The dietary recall section of the survey (Section 2) allowed participants to report whether and how often they consumed any of the following 13 broad beverage types in the past 24-h: bottled water, CSD, coffee, dairy-based drinks, energy drinks, flavored water, nutritional beverages, plain milk (consumed as a beverage), plant-based drinks, plant water, sports drinks, still juices and fruit-flavored drinks, and tea. ABA-BSW survey participants were asked to select any of the 13 beverage types listed above for each consumption occasion. These beverage types were presented to participants using the beverage type names paired with neutral images (see Supplemental File S1). The following information for each reported CO in Section 2 was gathered:

- Time of beverage consumption (6 a.m.–8 a.m., 8 a.m.–11 a.m., 11 a.m.–2 p.m., 2 p.m.–5 p.m., 5 p.m.–8 p.m., and 8 p.m.–6 a.m.),
- Type of beverage (e.g., regular CSD, LNCS CSD, ready-to-drink or not, caffeinated or decaffeinated, sparkling or still, etc.),
- Brand of beverage from a listing for each beverage type and an option to enter the brand (if not listed), and
- Container volume and fraction of the container consumed (e.g., between ½ and ¾ of a 20–24-ounce container); participants were also permitted to directly enter the volume consumed.

When a range was selected, the volume of each beverage in a CO was derived using the midpoint of both the container size and the fraction consumed. For example, if a participant reported consuming “between 3 quarters and the full container” of a 12–16 ounce (355–472 mL) container, the estimated consumption volume was calculated as:

$$\frac{0.75+1}{2}\;*\;\frac{355\;mL+472\;mL}{2}\;≌\;362\;mL\;$$

In the third and last section of the survey, participants were prompted to complete a 12-month FFQ collecting information on their annual consumption of 59 beverage subtypes within the 13 broad beverage types. For each subtype, participants were given the following 10 frequency option responses: “More than once per day”, “Once a day”, “4–6 times a week”, “2–3 times a week”, “Once a week”, “Once in 15 days”, “Once a month”, “Less than once a month”, “Once a year”, and “Never had”. Data on usual tap water consumption (typical daily amount and usual frequency of consumption) were also recorded in Section 3. 

Prior to launching, the survey instrument was pilot tested through a random sample of 100 BSW panel members to ensure data was correctly collated and that answers were logical. As a result of the pilot testing, the questions were modified to provide clearer definitions of the beverage types, additional clarifying beverage subtypes, and revisions to the brand selection options.

Panel members were invited to participate in the finalized ABA-BSW survey based on pre-defined geographic and socio-demographic quotas. Quotas were primarily guided by statistics from the U.S. Census Bureau, including age, sex, and race/ethnicity across different States [22]. All respondents received remuneration in the form of loyalty points, which can be redeemed through purchases across a variety of websites, post completion of the survey. BSW abided by the ICC/ESOMAR International Code on Market and Social Research in its development and administration of the survey. 

**Figure 1:**
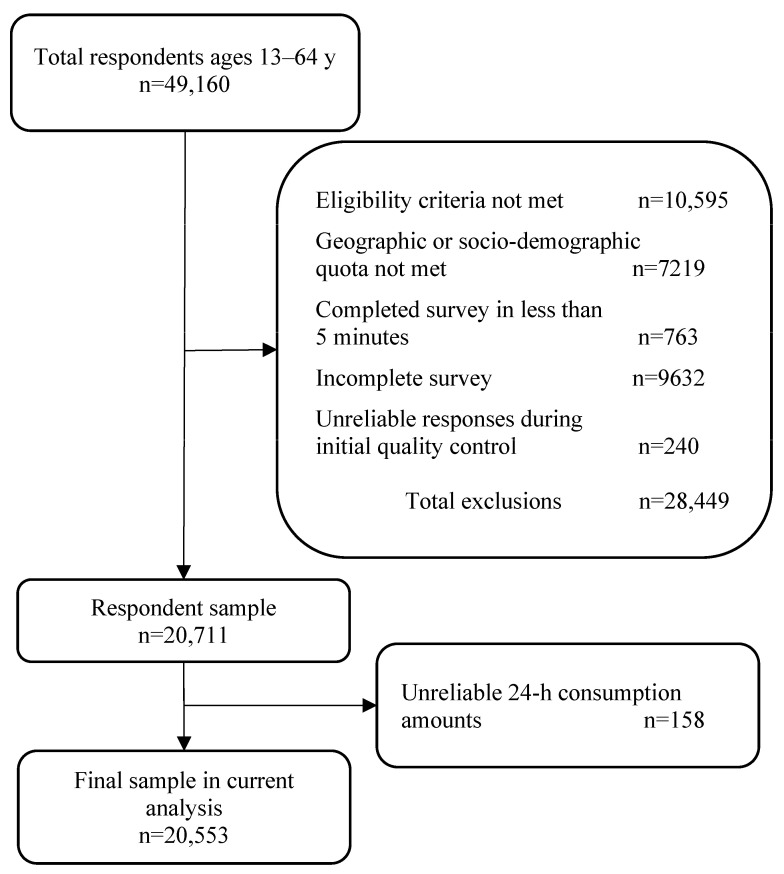
American Beverage Association-Brandscapes Worldwide survey (ABA-BSW) 2021 study population flow chart.

The number of total respondents in ABA-BSW was 49,160 (Figure 1). Of these, 28,449 were excluded by BSW because individual participants either did not meet the eligibility criteria or the pre-defined geographic or socio-demographic quota, did not complete the survey, completed the survey in less than 5 min, or their responses were identified as unreliable during initial quality control (e.g., illogical pairings of 24-h dietary recall reporting and 12-month FFQ responses). The resulting sample included 20,711 participants.

Statistical weights were derived using a random iterative method (RIM), also known as “raking” [23], that were assigned to survey respondents. The RIM corrects for sampling selection bias by ensuring that the weighted distribution of the sample is in close agreement with the target population’s marginal counts for select characteristics. Statistical weights for age, sex, and race/ethnicity in the ABA-BSW survey were based on 2019 State estimates derived by the U.S. Census Bureau from the 2010 decennial census [22]. Statistical weights for income were based on 2018 household income percentiles by State, which were derived from the Integrated Public Use Microdata Series, Current Population Survey [24]. 

### 2.2. WWEIA Component of NHANES

The NHANES provides information on the health and nutritional status of the non-institutionalized civilian resident population of the U.S. by using a complex, multistage, probability sampling design. Data collected by NHANES include demographic, health, and nutrition interviews and physical examinations, and are often used to estimate dietary intakes and the prevalence of various diseases and conditions. Approval for NHANES data collection was provided by the NCHS Research Ethics Review Board (Continuation of Protocol #2011-17 and Protocol #2018-01, 26 October 2017). Prior to the COVID-19 pandemic, the survey data were released in 2-year cycles, with participants surveyed across a calendar year. There was a lapse in field operations during the period from March 2020 to June 2021 due to the pandemic. NHANES field operations resumed in July 2021 to collect data for the NHANES 2021–2022 survey cycle. 

Dietary recall data are collected in NHANES using the Automated Multiple-Pass Method (AMPM) [25,26,27,28,29] to improve complete and accurate food recall among participants. The AMPM includes five steps. The first step collects a list of foods and beverages consumed the previous day. The second step probes for foods or beverages forgotten in the list. The third step collects the actual time of and CO name (e.g., lunch) for each food and beverage. The fourth step collects a detailed description for each food or beverage (e.g., caffeinated vs. decaffeinated, brand name, etc.), the amount consumed, and additions such as cereal with milk added or coffee with cream added. The fifth step consists of a final probe for anything else that may have been consumed. The NHANES dietary recall includes up to two days of intake data for each participant: the first-day data are collected in person and the second-day data are collected by telephone 3 to 10 days later, typically on a different day of the week. NCHS constructs and assigns statistical weights to each survey participant to adjust for unequal probability of selection, non-response, and the day of the week of the interview. Data from NHANES 2017–2018, the most recent available dietary recall data at the time of this analysis were used in the current study. 

### 2.3. Study Population

Since the ABA-BSW was an online questionnaire and therefore by design did not allow for immediate or in-person confirmation of the reported information (i.e., not an AMPM approach), respondents were excluded from the current study (*n* = 158) if their reported consumption amounts were identified unreliable as compared to the distribution of the consumption patterns observed from the NHANES 2017–2018 day 1 dietary recall (e.g., ABA-BSW respondents with more than 10 COs during any time interval were excluded based on observations of no more than six COs for any beverage during any of the six time intervals in NHANES). The identification and exclusion of unreliable 24-h consumption amounts in ABA-BSW are described in the Supplemental Methods. Therefore, the final ABA-BSW sample used for the current analysis was 20,553 (Figure 1). 

**Figure 2:**
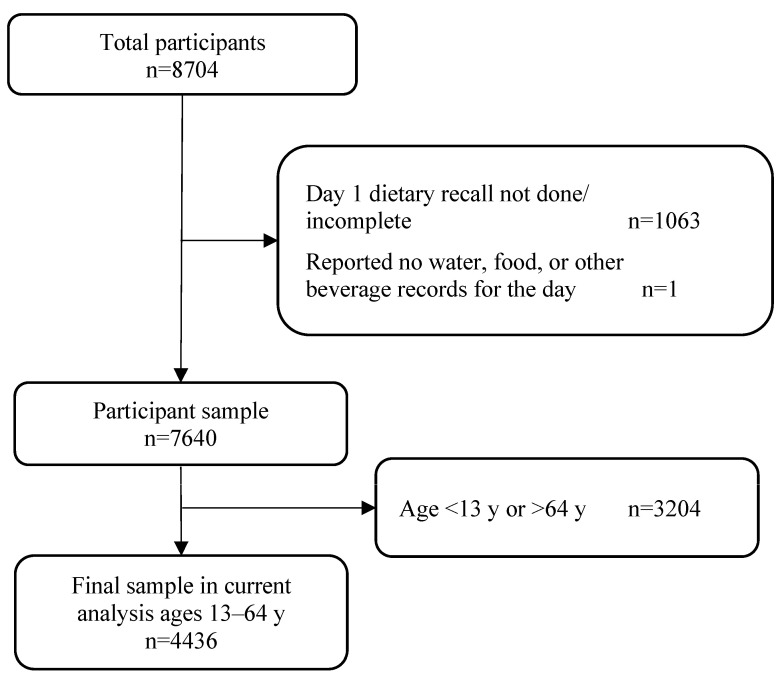
National Health and Nutrition Examination Survey (NHANES) 2017–2018 study population flow chart.

NHANES participants were restricted to those whose day 1 dietary recalls were deemed reliable and met the minimum criteria set by NCHS, which included completion of the first four steps of the five-step AMPM and identification of all foods consumed for each reported meal. Participants who completed the AMPM but did not report any consumption in the past 24-h were excluded. The sample was further restricted to participants ages 13 to 64 y to be directly comparable to the final ABA-BSW study population, resulting in an analytical sample of 4436 participants (Figure 2).

### 2.4. Statistical Analyses

Summary statistics were generated for participants in both surveys. The analysis was restricted to variables common to both surveys, specifically age, sex, race/ethnicity, marital status, and number of household members. 

The beverages and amounts reported consumed as a CO in the 24-h dietary recall of NHANES 2017–2018 were reviewed and mapped to the same 13 beverage types in ABA-BSW and further distinguishing between regular and LNCS CSD. A mapping summary of the 13 beverage types and corresponding NHANES beverages is provided in Table 1. A full listing of the NHANES codes is provided in Supplemental Table S1. NHANES CSD codes were separated into regular and LNCS in a similar manner. To align with the data collected for plain milk in ABA-BSW, plain milk COs and the corresponding reported amounts in NHANES were limited to those COs that were not reported in combination with any other food (e.g., milk and cereal, milk in coffee, etc.). In addition, the corresponding time of each NHANES CO was mapped to the broader intervals in ABA-BSW. The gram amounts of beverages reported consumed in NHANES were converted to milliliters, assuming a density of water (1 g/mL). 

Estimates of percent consumers, *per capita* mean, *per consumer* mean, and select percentiles (i.e., 10th, 25th, 50th, 75th, and 90th percentile) of the total daily consumption of each of the 13 beverage types were derived for both surveys. The 90th-to-10th percentile (P90:P10) ratio was used to compare the consumption variability in the surveys. In addition, the number of reported COs per day and the amount consumed per CO were summarized. Estimates were derived for the total sample of participants 13–64 y, as well as for three age group strata including 13–19 y, 20–49 y, and 50–64 y. Further, summary estimates per time interval within each beverage type were generated. 

No statistical hypothesis tests were conducted given that the survey designs were dissimilar. Further, the RIM sampling design for ABA-BSW was non-probabilistic, and therefore estimates of variance in this survey would likely be underestimated. However, the mean and percentile estimates allowed for a quantitative comparison of the consumption distributions resulting from each survey. All analyses were conducted in STATA v11.2. STROBE-nut guidelines were followed throughout [30].

## 3. Results

### 3.1. Demographic Characteristics

Table 2 summarizes the characteristics of the ABA-BSW and NHANES study populations. The two study populations had similar age, sex, and race/ethnicity weighted distributions. The ABA-BSW study population had a substantially higher proportion of single participants than in NHANES. The ABA-BSW study population also had a relatively lower proportion of participants from households with six or more members and a higher proportion from one- to three-member households. 

### 3.2. Total Daily Consumption Amounts

**Figure 3:**
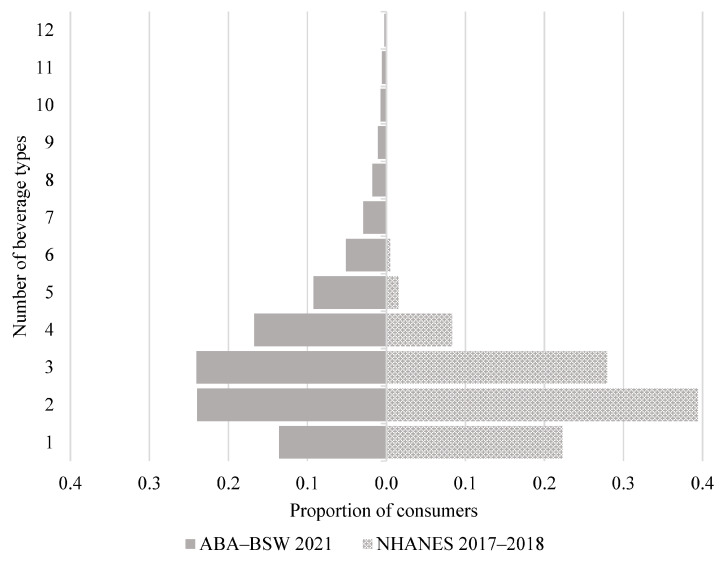
Number of beverage types consumed among consumers ages 13–64 y.

Table 3 summarizes the number of consumers, and the mean and distribution of the consumption amounts among consumers of the combined 13 broad beverage types as well as per beverage type for each survey. Estimates are provided for the total sample aged 13–64 y and for each of the three age subgroups (Supplemental Table S2). The tables also include consumption estimates for tap water, although these are not directly comparable between surveys since ABA-BSW estimates are based on typical daily amounts while NHANES estimates are based on 24-h dietary recalls. Figure 3 shows the number of beverage types consumed among consumers by survey.

The percentage of consumers of the combined 13 beverage types was comparable between the surveys, although *per consumer* mean daily consumption in ABA-BSW was higher than the mean consumption observed in NHANES (1903 mL/day vs. 1704 mL/day) with a relative difference of <12%. Among consumers of any beverage type, a wider range of beverage types were consumed by ABA-BSW participants. The percentage of consumers for each beverage type was generally higher in ABA-BSW as compared to NHANES. However, the percentage of consumers of bottled water was comparable between the surveys (48.5% vs. 50.8%). A higher percentage of consumer (>10%) for CSD, dairy-based beverages, energy drinks, flavored water, plain milk, and sports drinks was observed in ABA-BSW compared to NHANES. Within CSD, the percentage of consumers of regular CSD in ABA-BSW was 13.5% higher than that observed in NHANES, while LNCS CSD was 7.2% higher in ABA-BSW than that observed in NHANES.

The *per consumer* mean consumption amount of still juices and fruit-flavored drinks were comparable between surveys. On both a *per capita* and *per consumer* basis, the mean consumption amount of all CSD in ABA-BSW was more than that of NHANES (385 mL/day vs. 241 mL/day and 721 mL/day vs. 672 mL/day, respectively). Similarly, the mean consumption amounts of regular CSD and LNCS CSD were higher in ABA-BSW than in NHANES except for the *per consumer* mean of LNCS CSD (668 mL/day vs. 788 mL/day). Additionally, the *per consumer* consumption amount of all other beverage types except plant-based drinks was less in ABA-BSW than in NHANES. In contrast, the *per capita* mean consumption amounts were far more similar, and in some cases moderately higher in ABA-BSW than in NHANES. ABA-BSW participants reported consuming lower consumption amounts of bottled water when compared to NHANES (e.g., mean consumption amount of 428 mL/day vs. 692 mL/day *per capita* and 882 mL/day vs. 1362 mL/day *per consumer*). The distribution of consumption amounts across all beverage types among consumers in ABA-BSW showed relatively more variability, as measured by the P90:P10 ratio, than those observed in NHANES, with the exception of energy drinks and tap water. Excluding tap water, the consumption distribution of bottled water and energy drinks in NHANES were observed to have the largest variability (P90:P10 ratio = 8) across all beverage types, while plant-based drinks had the largest variability (P90:P10 ratio = 14) in ABA-BSW. The percentage of consumers of tap water in ABA-BSW was almost twice that observed in NHANES, while the mean daily amount of tap water was noticeably higher in NHANES than in ABA-BSW. 

Survey comparisons of the percentage of consumers and consumption amounts for the 13 beverage types (combined and individually) by age subgroup were generally similar as they were for the total sample aged 13–64 y. Among the 13–19 y subgroup, the percentage of consumers of bottled water, coffee, energy drinks, flavored water, and sports drinks in ABA-BSW was more than 10% higher than in NHANES. Among the 20–49 y subgroup, the percentage of consumers of CSD (regular and LNCS), dairy-based beverages, energy drinks, flavored water, plain milk, sports drinks, and still juice and fruit-flavored drinks in ABA-BSW was more than 10% higher than those observed in NHANES. In addition to CSD and plant-based drinks, which had substantially higher *per consumer* consumption amounts generally observed in ABA-BSW across adult subgroups compared to NHANES, flavored water also had substantially higher *per consumer* consumption amounts in ABA-BSW for the 13–19 y subgroup than in NHANES. Among the 50–64 y subgroup, CSD was the only beverage type where the percent consumer was more than 10% higher in ABA-BSW than in NHANES, with daily CSD consumption amounts higher in ABA-BSW than in NHANES.

### 3.3. Number of COs per Day and Amount per CO

Table 4 summarizes the number of COs per day and the average amount consumed per CO for each beverage type and all types combined. The average daily number of all COs reported by each participant in ABA-BSW was almost twice that of NHANES (6.1 COs/day vs. 3.3 COs/day). The average number of daily COs in ABA-BSW was higher than in NHANES for all beverage types except plant-based drinks and plant water, where the daily average number was similar across surveys. In contrast, the average amount consumed per CO was noticeably lower across beverage types except for plant-based drinks. 

Survey comparisons of the average number of daily COs and the amount consumed per CO by age subgroup were generally similar as they were for the total sample aged 13–64 y (Supplemental Table S3A–C). Among the 13–19 y and 50–64 y subgroups, the average number of daily COs in ABA-BSW was higher than in NHANES for all beverage types. The average number of daily COs in ABA-BSW among 20–49 y was marginally lower than in NHANES for plant-based drinks and plant water, but higher for all other beverage types. Similar to the total sample of 13–64 y, the average amount consumed per CO was lower in ABA-BSW compared to NHANES except for plain milk among the 20–49 y and 50–64 y subgroups. 

### 3.4. Consumption Patterns during the Day

**Figure 4:**
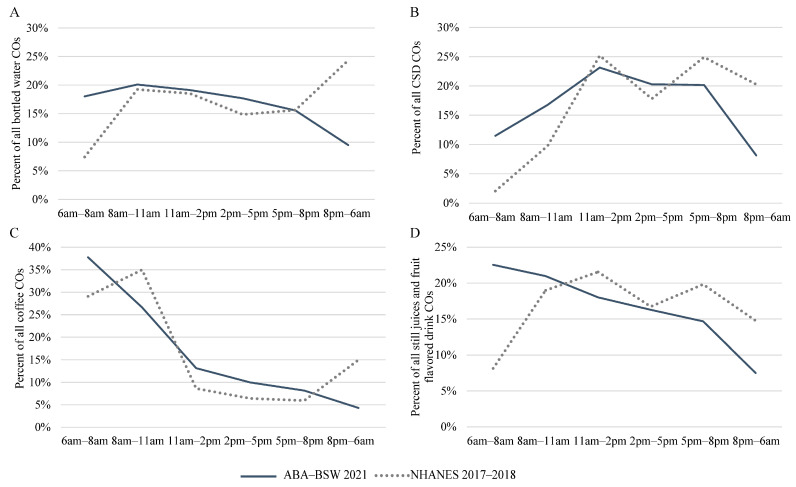
Percent distribution of beverage COs within six time intervals among consumer ages 13–64 y by survey; (**A**) bottled water, (**B**) CSD (total), (**C**) coffee, and (**D**) still juices and fruit-flavored drinks.

Figure 4 compares the percent distributions of the COs in the six time intervals between surveys for bottled water, CSD (total), coffee, and still juices and fruit-flavored drinks. Visualizations for the remaining beverage types and all beverage types combined are provided in Supplemental Figure S1. There was generally a higher percentage of COs occurring in the early morning interval (6 a.m.–8 a.m.) and a lower percentage of COs in the evening/overnight interval (8 p.m.–6 a.m.) across all beverage types in ABA-BSW as compared to NHANES. Large shifts in the percent distribution of COs across time intervals were observed in NHANES, though not in ABA-BSW. 

Sensitivity analyses that included the ABA-BSW sample prior to excluding those considered to have unrealistic consumption behaviors (*n* = 20,711) did not result in any noteworthy differences. 

## 4. Discussion

This preliminary analysis contextualizes and supports the findings from the 2021 ABA-BSW survey by comparing it with 2017–2018 consumption estimates from the established NHANES survey. The reported consumption patterns from ABA-BSW and NHANES were generally similar, with some differences noted. These similarities support the validity of the new survey and its value in future dietary investigations. Differences are likely due to the designs of the surveys, such as the geographic sampling frames, the method of implementation and data collection, the survey question structure, and the time periods during which each survey was conducted (i.e., during vs. pre-pandemic, respectively). 

### 4.1. Total Daily Consumption Amounts

While the total daily reported beverage consumption amount (excluding tap water) among consumers in ABA-BSW was generally higher than in NHANES, the difference in the mean was less than a cup (approximately 200 mL and <12% relative difference). The lower mean daily consumption amount and a higher percentage of consumers for most beverage types observed in ABA-BSW suggest that ABA-BSW participants are consuming a wider range of beverage types per day. This within-person dispersal of consumption across beverage types could lower the distribution of the amount consumed between persons in a single beverage type, which would explain the overall decrease in the mean daily amount consumed for several beverage types as compared to NHANES. This hypothesis is supported by the overall smaller differences observed between surveys at the *per capita* mean consumption for most beverage types. On the other hand, the observed differences in the combined beverage consumption and across types may also reflect a true shift in consumption patterns between the two survey time periods, either because of the availability of more beverage types in 2021 as compared to 2017–2018 or because of a shift in dietary patterns due to the COVID-19 pandemic. Mignogna et al. [31] conducted a systematic review of the literature comparing dietary habits before and during the pandemic and found that 81% of the 59 articles reporting changes in consumption behaviors found increases in food consumption. A report by marketing research companies Zippia and Grand View Research showed an 8 to 12% increase in sales of non-alcoholic beverages between 2017–2018 and 2021 [32], with beverage companies responding with product expansion and the launch of new product lines to meet higher consumer demand [32]. 

A larger percentage of CSD (total, including both regular CSD and LNCS CSD) was observed in ABA-BSW when compared to NHANES and was paired with an increase in the *per consumer* total CSD mean consumption amounts, driven by higher regular CSD consumption amounts. Studies conducted early on during the pandemic identified significantly increased consumption of sugar-sweetened beverages [31,33]. 

Other studies have also documented changes in consumption patterns during the COVID-19 pandemic. In an online survey conducted by Chenarides et al. [34] with 693 participants, 32% reported an increase in bottled water consumption (plain, flavored, etc., not discernable) while 14% reported a decrease. On the other hand, in a study conducted by Bin Zarah et al. [35] that assessed whether the consumption of select foods and beverages by 3133 adults increased, decreased, or remained unchanged during the COVID-19 pandemic, more survey participants reported an increase in water consumption than a decrease (35% vs. 11%) [35]. In the current study, the percentage of consumers of bottled and/or flavored water combined in ABA-BSW was relatively unchanged as compared to NHANES (55.8% vs. 52.1%, respectively), with the daily mean consumption amounts of bottled and flavored water combined among consumers being lower in ABA-BSW as compared to NHANES (932 mL vs. 1357 mL). 

Bin Zarah et al. [35] reported a higher percentage of adults with an increase in coffee and/or tea consumption than a decrease (31% vs. 10%) during COVID-19. In the current study, a higher percentage of consumers for coffee and/or tea combined was observed in ABA-BSW as compared to NHANES (67.3% vs. 63.4%), although mean consumer consumption amounts were lower in ABA-BSW (650 mL vs. 701 mL). Similarly, the Bin Zarah et al. study [35] also reported a higher percentage of adults with an increase in regular and LNCS CSD than a decrease. In the current study, ABA-BSW had a higher percentage of CSD consumers (total, regular CSD, and LNCS CSD), though only the amount consumed for regular CSD (i.e., not LNCS CSD) was higher than compared to NHANES. The overall larger relative variability of the daily amounts consumed across beverage types in ABA-BSW as compared to NHANES is likely a reflection of the larger ABA-BSW sample size, which allowed for far more observations and a larger distribution.

The higher percentage of consumers and lower mean consumption of tap water in ABA-BSW compared to NHANES was likely the result of survey responses that are not directly comparable. ABA-BSW asked participants about average daily tap water consumption during the past year, while NHANES estimates were based on tap water consumption reported from the past 24-h. It has been shown that as the survey duration lengthens, the percentage of consumers increases while the mean *per consumer* amount decreases [36], which may support the differences observed for tap water. 

### 4.2. Number of COs per Day and Amount per CO

Despite the overall similarities in daily consumption amounts between the surveys, higher frequencies of COs per day and lower amounts consumed per CO were observed in ABA-BSW as compared to NHANES. This conflicting pattern is likely driven by the differences in the survey interface. Specifically, providing participants with a series of time intervals to record their beverage COs and asking them to report consumption by using fractions of a container may have led ABA-BSW survey participants to report multiple COs when consumption of a single beverage spanned two or more intervals. The splitting of these occasions would therefore also lower the consumption amount per CO. In contrast, NHANES asks participants to name each CO (e.g., breakfast), therefore grouping COs into longer time intervals and thus reducing the opportunity for split COs. Further, some of the COs in NHANES that spanned over more than one named CO were classified as “extended”, a non-overlapping category, which could have resulted in even higher consumption amounts per CO and fewer COs per day. 

The difference between the two surveys with respect to the number of COs could also be due to a change in consumption patterns during the pandemic. Mignogna et al. [31] also found that 67% and 87% of the articles reporting changes in consumption behaviors observed increases in the number of meals per day and snacking, respectively. 

A shift to smaller packaged beverage products by consumers may also contribute to higher COs and lower amounts consumed per CO in ABA-BSW compared to NHANES. In a qualitative study aimed to explore consumers’ experiences of consuming cola from smaller bottles compared with larger bottles, the smallest bottles were perceived to increase drinking occasion frequency and encourage consumption of numerous bottles in succession [37]. Further exploration of the ABA-BSW dataset to assess the impact of smaller bottles on the actual consumption patterns of beverages is recommended. 

Large shifts in the percentage of distribution of COs across time intervals observed in NHANES and not in ABA-BSW are likely driven in part by the smaller number of COs for some beverage groups. Further, NHANES participants report the actual time of each CO, most often on the hour. The mapping of NHANES COs to the ABA-BSW time intervals could, therefore, also contribute to measurement error, leading to certain time intervals with a greater proportion of COs simply as a result of being reported at the top of the hour (e.g., 11 am). 

### 4.3. Comparing the Design of the the ABA-BSW 2021 and NHANES 2017–2018 Surveys (Strengths and Limitations)

A major strength of this study is that it introduced and validated the ABA-BSW dataset, which adds valuable information on recent U.S. beverage consumption patterns. While NHANES is also statistically weighted to be representative of the entire U.S. non-institutionalized civilian population, its design inherently restricts its sampled population to specific geographic regions and communities, which are not publicly available. In contrast, ABA-BSW sampled from individuals across the U.S., providing important information on the geographic distribution of consumption patterns. Another strength of the ABA-BSW survey is that it was an iterative questionnaire that probed participants to enter detailed information on each CO, including the beverage type, brand, time interval of consumption, and the amount consumed. In addition, ABA-BSW included an FFQ that allows the frequency of consumption and gram of intake per CO to be linked for the same population, thus allowing for the derivation of empirical estimates of usual intakes in future studies. NHANES has not conducted an FFQ on beverage consumption since 2006, and so the frequency data collected in the ABA-BSW survey provides a unique opportunity to estimate long-term beverage consumption patterns in the current era. 

The increased number of COs in the ABA-BSW 24-h survey as compared to NHANES 24-h dietary recall may provide new opportunities to gain insight into consumption patterns in a single day. This will thus allow for analyses not possible in other surveys such as NHANES. For example, the ABA-BSW CO data could allow for making a distinction between individuals who consume a single beverage in a short period of time and those sipping a beverage throughout the day. This could be further investigated by comparing the consistency of brands consumed by a single survey participant across their reported COs. 

ABA-BSW and NHANES have limitations common to all cross-sectional recall surveys in that they provide a snapshot of consumption and the data are subject to recall bias and potential underreporting. ABA-BSW was limited to beverage consumption for the 13–64 y age group primarily because its main objective was to focus on the young adult and adult beverage consuming population with a secondary objective to assess caffeine intake estimates among these age groups. Previous studies have shown that caffeinated beverages are the primary source of caffeine intake in the U.S. [38,39]. Caffeine intakes for children 12 y or less and adults 65 y or older are lower than those observed among younger adults [16,40], and for adults 65 y or older, lower than the 50–64 y age group. 

A limitation of the ABA-BSW survey is that it was conducted in September-November 2021, and therefore may not be representative of consumption patterns during other parts of the year and also may not account for seasonal variations. In contrast, NHANES is continuously sampled over the year, and so accounts for temporal variation in consumption patterns. Another potential limitation of ABA-BSW is the use of a self-administered questionnaire with no interviewer to guide the respondents, or confirm that no COs were missed, or perform immediate checks on potentially non-sensical responses. However, in the absence of an interviewer, BSW incorporated screening criteria and quality control processes to optimize the robustness of the collected data.

### 4.4. Additional Comments

The distributions of the estimated daily total amount of beverage consumed were generally comparable between the two surveys, supporting the validity of the ABA-BSW dataset. The observed differences in consumption amounts and number of COs between the two samples are more likely the result of changes in consumption patterns associated with the pandemic and/or the increased availability of certain products (e.g., plant water and plant-based drinks) in the U.S. market. This can be corroborated in the future when NHANES 2021–2022 dietary data that covers the ABA-BSW survey period (i.e., September to November 2021) are publicly released. NHANES data from the 2019–2020 cycle of the survey, in combination with the 2017–2018 cycle data, were released by NCHS in July 2021. This release is unique as the 2019–2020 data alone are not considered to be nationally representative given that field operations were suspended in March 2020 due to the COVID-19 pandemic. Recent studies [31,34,35,41] have indicated that dietary patterns have been affected by the pandemic and associated lockdowns. Until NHANES 2021–2022 dietary data are released, the ABA-BSW dataset has likely collected the richest source of nationally representative beverage consumption data in the U.S. since the beginning of the pandemic, an unintentional strength of the survey. 

Given the large time difference between the ABA-BSW 2021 and NHANES 2003–2006 FFQs, a comparison was not relevant to the current study. Such a comparison may be worth undertaking at a later time, but only if considering additional, more recent sources of beverage consumption frequency.

Inferential statistics were not performed in this study, as the quota sampling design for the ABA-BSW survey is non-probabilistic, which can result in underestimates of variance. Even if inferential statistics for ABA-BSW were conducted, the difference in survey structures would prohibit conducting informative hypothesis tests. The use of descriptive statistics, however, still provides valuable information on the similarities and differences in U.S. beverage consumption patterns between the two surveys.

The differences between the demographic make-up of the two surveys, specifically the proportion of household size and participants identifying as “single”, could have contributed to some of the differences observed in consumption patterns across beverage types. Based on household purchase data from the 2000–2010 Nielsen Homescan Panel, Piernas et al. [42] reported that among consumers of sugar-sweetened beverages, households with children had a higher average number of SSB servings per day than households without children (1.35 servings/day vs. 0.80–1.06 servings/day). In comparison, the average number of LNCS beverage servings per day among households with children was within the range of households without children (1.30 servings/day vs. 1.13–1.35 servings/day) [42]. Capps et al. [43] found a positive association between household size and the probability of purchasing non-alcoholic beverages (i.e., fruit juice and drinks, sports drinks, powdered soft drinks, coffee and tea, CSD, bottled water, and milk). The current analyses did not adjust for the observed differences in household size and marital status for either the ABA-BSW or NHANES surveys. 

## 5. Conclusions

The present study compared consumption patterns reported in ABA-BSW, which collected data through an online survey on beverage consumption in the previous 24-h among a nationally representative sample of the U.S. population aged 13–64 y, to those reported in NHANES 2017–2018. The patterns were generally similar, increasing confidence in the consumption data collected in the ABA-BSW dataset. The noted differences may be due to the distinct survey designs, reflect a shift as a result of changes in dietary patterns during the COVID-19 pandemic, and/or increased products and availability in 2021. The ABA-BSW dataset also provides additional information on the within-day temporal beverage consumption patterns among a larger geographic swath of the U.S. population. The ABA-BSW survey provides a rich new dataset with which to conduct more nuanced analyses on the intake of beverage ingredients, such as caffeine and artificial sweeteners, among the U.S. population aged 13–64 y. This study supports the validity of the ABA-BSW survey, serving as a foundation for these future analyses. 

## Figures and Tables

**Table 1:** Examples of NHANES Food Codes mapped to ABA-BSW Beverage Types.

Beverage Types	Description/Examples
Bottled water	Unsweetened bottled still or sparkling water
Carbonated Soft drink (CSD)	Regular, diet, reduced sugar, caffeinated or caffeine-free carbonated soft drinks such as but not limited to ginger ale, cola, root beer, and pepper type
Coffee	Hot or iced brewed, instant, or bottled coffee including but not limited to with/without milk, with/without sweetener, with/without caffeine café con leche, cappuccino, latte, Turkish coffee, cafe mocha, and other coffee drinks
Dairy-Based Drinks	Chocolate and other flavored milk (includes hot chocolate/cocoa), eggnog, malted milk, dairy-based smoothie, milkshake
Energy Drinks	Sugar-sweetened and sugar-free energy drinks including but not limited to Red Bull, Rockstar, and Monster
Flavored Water	Sugar-sweetened or low-calorie or no-calorie sweetened flavored water beverages including but not limited to Glaceau Water and Propel
Nutritional Beverages	Meal supplement or replacement beverages including but not limited to Boost, Ensure, and other protein drinks; nutritional powder converted to reconstituted volumes
Plain Milk consumed as a beverage	Plain cow’s milk and goat’s milk, whole, low fat, reduced fat, fat free
Plant-Based Drinks	Almond milk, soy milk, rice milk, and coconut milk including but not limited to flavored drinks
Plant water	Coconut water
Sports Drinks	Thirst quencher beverage including but not limited to Gatorade and Powerade
Still Juices and Fruit Flavored Drinks	100% fruit and vegetable juices, fruit nectar, fruit and/or vegetable based smoothies, and fruit-flavored drinks
Tap water	Tap Water
Tea	Hot or iced brewed, instant, or bottled teas including but not limited to unsweetened, pre-sweetened, regular or diet black, green, and herbal teas

**Table 2:** Population characteristics of ABA-BSW and NHANES 2017–2018 sample ages 13–64 y.

Characteristic, Weighted %	Survey	
	ABA-BSW 2021	NHANES 2017–2018
Unweighted Sample Size	20,553	4436
Age (y) ^1^	38 (25, 51)	38 (25, 52)
Age Group		
13 to 19 y	13.0%	13.7%
20 to 49 y	58.5%	56.5%
50 to 64 y	28.5%	29.9%
Sex		
Male	49.8%	49.0%
Female	50.2%	51.0%
Race/Ethnicity		
Hispanic	19.1%	18.8%
Non-Hispanic, White	58.6%	56.9%
Non-Hispanic, Black	13.1%	12.5%
Non-Hispanic, Asian	6.3%	6.3%
Other race	3.0%	5.6%
Marital Status ^2^		
Single	38.6%	23.9%
Married/In a relationship	51.6%	61.5%
Divorced/Widowed/Separated	8.9%	14.6%
Declined to Answer	0.9%	0.04%
Household size		
1 to 3	63.5%	55.2%
4 to 5	31.1%	33.6%
6 or more	5.4%	11.3%

^1^ Median (Quartile 1, Quartile 3). ^2^ Marital status for NHANES sample restricted to those aged 20+ y.

**Table 3:** Daily consumption of all beverage types combined and individual beverage types among ages 13–64 y.

Beverage Type	Statistics (mL/Day)	Survey	
		ABA-BSW 2021	NHANES 2017–2018
		(*n* = 20,553)	(*n* = 4436)
All beverages (excluding tap water)	% Consumers	98.7%	97.0%
	Per Capita Mean	1878	1653
	Per Consumer Estimates		
	Mean	1903	1704
	P10	474	445
	P25	896	805
	P50	1577	1431
	P75	2510	2226
	P90	3646	3240
	P90:P10 ratio	8	7
Bottled water	% Consumers	48.5%	50.8%
	Per Capita Mean	428	692
	Per Consumer Estimates		
	Mean	882	1362
	P10	181	360
	P25	361	507
	P50	631	1014
	P75	1241	1800
	P90	2022	2700
	P90:P10 ratio	11	8
CSD (total)	% Consumers	53.4%	35.8%
	Per Capita Mean	385	241
	Per Consumer Estimates		
	Mean	721	672
	P10	134	186
	P25	293	334
	P50	536	480
	P75	943	782
	P90	1538	1326
	P90:P10 ratio	11	7
Regular CSD	% Consumers	43.5%	30.0%
	Per Capita Mean	294	190
	Per Consumer Estimates		
	Mean	676	634
	P10	125	186
	P25	260	326
	P50	476	450
	P75	887	744
	P90	1478	1256
	P90:P10 ratio	12	7
Low- and no-calorie sweetened (LNCS) CSD	% Consumers	13.6%	6.4%
	Per Capita Mean	91	50
	Per Consumer Estimates		
	Mean	668	788
	P10	114	240
	P25	260	360
	P50	414	507
	P75	874	960
	P90	1478	1440
	P90:P10 ratio	13	6
Coffee	% Consumers	54.5%	47.4%
	Per Capita Mean	271	263
	Per Consumer Estimates		
	Mean	497	555
	P10	125	195
	P25	236	270
	P50	355	390
	P75	623	675
	P90	1006	1020
	P90:P10 ratio	8	5
Dairy-based beverages	% Consumers	14.9%	4.9%
	Per Capita Mean	48	20
	Per Consumer Estimates		
	Mean	326	406
	P10	79	176
	P25	125	248
	P50	255	352
	P75	414	494
	P90	634	636
	P90:P10 ratio	8	4
Energy drinks	% Consumers	20.1%	3.5%
	Per Capita Mean	85	20
	Per Consumer Estimates		
	Mean	426	564
	P10	114	124
	P25	181	360
	P50	338	496
	P75	536	744
	P90	830	960
	P90:P10 ratio	7	8
Flavored water	% Consumers	15.5%	2.2%
	Per Capita Mean	92	15
	Per Consumer Estimates		
	Mean	597	663
	P10	125	180
	P25	260	435
	P50	419	525
	P75	792	870
	P90	1241	1110
	P90:P10 ratio	10	6
Nutritional beverages	% Consumers	9.7%	4.6%
	Per Capita Mean	36	20
	Per Consumer Estimates		
	Mean	372	433
	P10	79	213
	P25	163	240
	P50	296	384
	P75	414	512
	P90	670	640
	P90:P10 ratio	9	3
Plain milk	% Consumers	23.8%	10.0%
	Per Capita Mean	89	38
	Per Consumer Estimates		
	Mean	375	384
	P10	79	153
	P25	159	244
	P50	273	336
	P75	414	488
	P90	754	641
	P90:P10 ratio	10	4
Plant-based drinks	% Consumers	9.9%	4.4%
	Per Capita Mean	32	10
	Per Consumer Estimates		
	Mean	323	223
	P10	48	61
	P25	125	92
	P50	227	214
	P75	390	320
	P90	650	381
	P90:P10 ratio	14	6
Plant water	% Consumers	1.7%	0.6%
	Per Capita Mean	8	3
	Per Consumer Estimates		
	Mean	443	579
	P10	79	300
	P25	181	444
	P50	375	690
	P75	536	690
	P90	852	690
	P90:P10 ratio	11	2
Sports drinks	% Consumers	19.7%	4.7%
	Per Capita Mean	101	31
	Per Consumer Estimates		
	Mean	512	670
	P10	114	248
	P25	204	372
	P50	414	620
	P75	650	870
	P90	1072	992
	P90:P10 ratio	9	4
Still juices and fruit flavored drinks	% Consumers	34.2%	25.3%
	Per Capita Mean	160	118
	Per Consumer Estimates		
	Mean	469	466
	P10	114	140
	P25	181	233
	P50	328	357
	P75	588	543
	P90	984	870
	P90:P10 ratio	9	6
Tea	% Consumers	33.1%	27.7%
	Per Capita Mean	167	182
	Per Consumer Estimates		
	Mean	504	656
	P10	118	195
	P25	224	300
	P50	355	512
	P75	600	805
	P90	1065	1320
	P90:P10 ratio	9	7
Tap water ^1^	% Consumers	81.8%	46.8%
	Per Capita Mean	828	655
	Per Consumer Estimates		
	Mean	1012	1400
	P10	250	304
	P25	375	525
	P50	750	1035
	P75	1250	1920
	P90	1750	2899
	P90:P10 ratio	7	10

^1^ Estimates for tap water not directly comparable between surveys as NHANES estimates are based on 24-h dietary recalls while ABA-BSW estimates on tap water are based on typical daily amounts from a food-frequency questionnaire (FFQ; tap water is not an option to select in the 24-h dietary recall online survey).

**Table 4:** Beverage consumption occasions (COs) among ages 13–64 y.

Beverage Type	ABA-BSW 2021			NHANES 2017–2018		
	Total Number of COs ^1^	Average		Total Number of COs	Average	
		COs/Day	mL/CO		COs/Day	mL/CO
All beverages (excluding tap water)	124,511	6.1	313	14,295	3.3	511
Bottled water	24,574	2.5	349	4798	2.0	685
CSD (total)	24,235	2.2	334	2366	1.5	460
Regular CSD	19,155	2.1	328	2036	1.5	449
LNCS CSD	5080	1.9	358	330	1.4	510
Coffee	19,285	1.7	291	2253	1.3	441
Dairy-based drinks	3897	1.3	249	275	1.1	370
Energy drinks	5915	1.4	304	126	1.1	526
Flavored water	6241	1.9	307	88	1.4	482
Nutritional beverages	2744	1.4	272	155	1.2	358
Plain milk	6619	1.4	269	509	1.1	336
Plant-based drinks	2641	1.3	246	194	1.3	171
Plant water	497	1.4	310	31	1.5	392
Sports drinks	5666	1.4	359	250	1.1	606
Still juices and fruit flavored drinks	10,773	1.6	298	1674	1.3	371
Tea	11,424	1.7	303	1576	1.4	483

^1^ Unweighted number of COs; all other statistics are appropriately weighted using survey weights.

## Data Availability

Data described in the manuscript, code book, and analytic code will be made available upon request, with the exception of the ABA-BSW data due to its proprietary nature.

## Supplementary Materials

The following supporting information can be downloaded at: https://www.mdpi.com/article/10.3390/nu15163561/s1, Supplemental Figure S1. Percentage of distribution of beverage COs within six time intervals among consumer ages 13–64 y by survey; (1A) all beverage types combined, (1B) Regular CSD, (1C) LNCS CSD, (1D) dairy-based drinks, (1E) energy drinks, (1F) flavored water, (1G) nutritional beverages, (1H) plain milk, (1I) plant-based drinks, (1J) plant water, (1K) sport drinks, and (1L) tea; Supplemental Table S1. Full list of NHANES eight-digit food codes mapped to thirteen Beverage Types; Supplemental Table S2. Consumers and daily intakes (mL) for all beverage types combined and by beverage type among subgroups 13–19 y, 20–49 y, and 50–64 y; Supplemental Table S3A. Beverage COs among ages 13–19 y, Supplemental Table S3B. Beverage COs among ages among ages 20–49 y, Supplemental Table S3C. Beverage COs among ages among ages 50–64 y.

## Abbreviations

ABA	American Beverage Association
AMPM	Automated Multiple-Pass Method
BSW	Brandscapes Worldwide
CO	Consumption occasion
CSD	Carbonated Soft Drinks
FFQ	Food frequency questionnaire
LNCS	Low- and No-Calorie Sweetened
NCHS	National Center for Health Statistics
NCI	National Cancer Institute
NHANES	National Health and Nutrition Examination Survey
P90:P10	90th-to-10th percentile ratio
RIM	Random iterative method
SSB	Sugar Sweetened Beverages
U.S.	United States
WWEIA	What We Eat in America
y	Years
