# Supplementary material for: Beverage Consumption Patterns among U.S. Adolescents and Adults from a New 24-h Beverage Recall Survey Compared to the National Health and Nutrition Examination Survey (NHANES) 2017–2018
Source: Nutrients. 2023 Aug 12;15(16):3561. doi: 10.3390/nu15163561 (PMC10459501; doi:10.3390/nu15163561)
Supplement: Supplementary file 1 [file nutrients-15-03561-s001.zip › nutrients-2446004-supplementary.pdf]

Xiaoyu Bi -- Beverage consumption patterns among U.S. adolescents and adults from a 24-hour dietary recall survey conducted in 2021 compared to the National Health and Nutrition Examination Survey (NHANES) 2017-2018

Supplemental Methods

Identification of outliers in (or questionable entries from) the 24-hr consumption in ABA-BSW

Since ABA-BSW was an online questionnaire and therefore by design did not allow for immediate or in-person confirmation of the reported information (i.e., it was not an AMPM approach), respondents were excluded from the current study if their reported intakes were deemed unreliable as compared to the distribution of the consumption patterns observed from the NHANES 2017-2018 day 1 dietary recall. The total daily intake of the 13 beverage types in NHANES ranged from 16 mL to 12728 mL when excluding tap water and 60 mL to 15696 mL when including tap water. There were no more than six COs for any beverage during any of the six time intervals. Since ABA-BSW included a larger number of participants than in the NHANES subsample of participants ages 13-64 y, it is possible that both lower and higher total daily intakes and number of COs per time interval could be observed in ABA-BSW as compared to the minimums and maximums observed in NHANES. Therefore, ABA-BSW survey participants whose total daily beverage amount (excluding tap water) exceeded 16000 mL or who had more than 10 COs during any time interval were identified as having potentially unreliable dietary recalls and were excluded from the current analysis. Less than 1% (n=158) of the 20,711 participants were excluded due to: exceedance of total daily volume (n=12), exceedance of COs/interval (n=122), and exceedance of both (n=24). The final ABA-BSW survey sample used for the current analysis was therefore 20,553.

Supplemental Table S1. Full list of NHANES eight-digit food codes mapped to thirteen Beverage Types

**Beverage Type**	**NHANES Food Code (8-digit and Name)**
**Bottled water**	92410210 Carbonated water, unsweetened
	94100100 Water, bottled, unsweetened
**Carbonated Soft drink**	92400000 Soft drink, NFS
	92400100 Soft drink, NFS, diet

	92410310	Soft drink, cola
	92410315	Soft drink, cola, reduced sugar
	92410320	Soft drink, cola, diet
	92410330	Soft drink, cola-type, with higher caffeine
	92410340	Soft drink, cola, decaffeinated
	92410350	Soft drink, cola, decaffeinated, diet
	92410360	Soft drink, pepper type
	92410370	Soft drink, pepper type, diet
	92410390	Soft drink, pepper type, decaffeinated
	92410400	Soft drink, pepper type, decaffeinated, diet
	92410410	Soft drink, cream soda
	92410420	Soft drink, cream soda, diet
	92410510	Soft drink, fruit flavored, caffeine free
	92410520	Soft drink, fruit flavored, diet, caffeine free
	92410550	Soft drink, fruit flavored, caffeine containing
	92410560	Soft drink, fruit flavored, caffeine containing, diet
	92410610	Soft drink, ginger ale
	92410620	Soft drink, ginger ale, diet
	92410710	Soft drink, root beer
	92410720	Soft drink, root beer, diet
	92410810	Soft drink, chocolate flavored
	92410820	Soft drink, chocolate flavored, diet
	92411510	Soft drink, cola, fruit or vanilla flavored
	92411610	Soft drink, cola, fruit or vanilla flavored, diet
	92416010	Mavi drink
	92417010	Soft drink, ale type
	92431000	Carbonated juice drink, NS as to type of juice
	92432000	Fruit juice drink, citrus, carbonated
	92433000	Fruit juice drink, non citrus, carbonated
**Coffee**	11561000	Cafe con leche
	11561010	Cafe con leche prepared with sugar
	92100000	Coffee, NS as to type
	92100500	Coffee, NS as to brewed or instant
	92101000	Coffee, brewed
	92101500	Coffee, brewed, blend of regular and decaffeinated
	92101600	Coffee, Turkish
	92101610	Coffee, espresso
	92101630	Coffee, espresso, decaffeinated
	92101700	Coffee, brewed, flavored
	92101800	Coffee, Cuban
	92101810	Coffee, macchiato
	92101820	Coffee, macchiato, sweetened

	92101850 Coffee, cafe con leche
	92101851 Coffee, cafe con leche, decaffeinated
	92101900 Coffee, Latte
	92101901 Coffee, Latte, nonfat
	92101903 Coffee, Latte, with non-dairy milk
	92101904 Coffee, Latte, flavored
	92101905 Coffee, Latte, nonfat, flavored
	92101906 Coffee, Latte, with non-dairy milk, flavored
	92101910 Coffee, Latte, decaffeinated
	92101911 Coffee, Latte, decaffeinated, nonfat
	92101917 Coffee, Latte, decaffeinated, flavored
	92101918 Coffee, Latte, decaffeinated, nonfat, flavored
	92101920 Frozen coffee drink
	92101921 Frozen coffee drink, nonfat
	92101923 Frozen coffee drink, with non-dairy milk
	92101925 Frozen coffee drink, with whipped cream
	92101926 Frozen coffee drink, nonfat, with whipped cream
	92101930 Frozen coffee drink, decaffeinated
	92101931 Frozen coffee drink, decaffeinated, nonfat
	92101935 Frozen coffee drink, decaffeinated, with whipped cream
	92101936 Frozen coffee drink, decaffeinated, nonfat, with whipped cream
	92101950 Coffee, Cafe Mocha
	92101955 Coffee, Cafe Mocha, nonfat
	92101960 Coffee, Cafe Mocha, with non-dairy milk
	92101965 Coffee, Cafe Mocha, decaffeinated
	92101970 Coffee, Cafe Mocha, decaffeinated, nonfat
	92101975 Coffee, Cafe Mocha, decaffeinated, with non-dairy milk
	92102000 Frozen mocha coffee drink
	92102010 Frozen mocha coffee drink, nonfat
	92102020 Frozen mocha coffee drink, with non-dairy milk
	92102030 Frozen mocha coffee drink, with whipped cream
	92102040 Frozen mocha coffee drink, nonfat, with whipped cream
	92102050 Frozen mocha coffee drink, with non-dairy milk and whipped cream
	92102090 Frozen mocha coffee drink, decaffeinated, with whipped cream
	92102400 Iced Coffee, brewed
	92102401 Iced Coffee, brewed, decaffeinated
	92102450 Iced Coffee, pre-lightened and pre-sweetened
	92102500 Coffee, Iced Latte
	92102501 Coffee, Iced Latte, nonfat
	92102502 Coffee, Iced Latte, with non-dairy milk
	92102503 Coffee, Iced Latte, flavored
	92102505 Coffee, Iced Latte, with non-dairy milk, flavored

	92102600 Coffee, Iced Cafe Mocha
	92102602 Coffee, Iced Cafe Mocha, with non-dairy milk
	92103000 Coffee, instant, reconstituted
	92104000 Coffee, instant, 50% less caffeine, reconstituted
	92105010 Coffee, made from liquid concentrate
	92106000 Coffee, acid neutralized, from powdered instant
	92111000 Coffee, NS as to brewed or instant, decaffeinated
	92111010 Coffee, brewed, decaffeinated
	92114000 Coffee, instant, decaffeinated, reconstituted
	92121000 Coffee, instant, pre-lightened and pre-sweetened with sugar, reconstituted
	92121001 Coffee, instant, decaffeinated, pre-lightened and pre-sweetened with sugar, reconstituted
	92121010 Coffee, instant, pre-sweetened with sugar, reconstituted
	92121020 Coffee, mocha, instant, pre-lightened and pre-sweetened with sugar, reconstituted
	92121030 Coffee, mocha, instant, pre-lightened and pre-sweetened with low calorie sweetener, reconstituted
	92121040 Coffee, instant, pre-lightened and pre-sweetened with low calorie sweetener, reconstituted
	92121041 Coffee, instant, decaffeinated, pre-lightened and pre-sweetened with low calorie sweetener, reconstituted
	92121050 Coffee, mocha, instant, decaffeinated, pre-lightened and pre-sweetened with low calorie sweetener, reconstituted
	92130000 Coffee, pre-lightened and pre-sweetened with sugar
	92130001 Coffee, decaffeinated, pre-lightened and pre-sweetened with sugar
	92130005 Coffee, pre-lightened and pre-sweetened with low calorie sweetener
	92130006 Coffee, decaffeinated, pre-lightened and pre-sweetened with low calorie sweetener
	92130010 Coffee, pre-lightened
	92130011 Coffee, decaffeinated, pre-lightened
	92130020 Coffee, pre-sweetened with sugar
	92130021 Coffee, decaffeinated, pre-sweetened with sugar
	92130030 Coffee, pre-sweetened with low calorie sweetener
	92130031 Coffee, decaffeinated, pre-sweetened with low calorie sweetener
	92152000 Coffee and chicory, brewed
	92153100 Coffee, decaffeinated, with cereal
	92161000 Coffee, Cappuccino
	92161001 Coffee, Cappuccino, nonfat
	92161002 Coffee, Cappuccino, with non-dairy milk
	92161005 Cappuccino, sweetened
	92162000 Coffee, Cappuccino, decaffeinated
	92162001 Coffee, Cappuccino, decaffeinated, nonfat
	92162005 Cappuccino, decaffeinated, sweetened

	92171000 Coffee, bottled/canned
	92171010 Coffee, bottled/canned, light
	92191000 Coffee, dry instant powder, NS as to regular or decaffeinated
	92191100 Coffee, instant, not reconstituted
	92191200 Coffee, instant, decaffeinated, not reconstituted
	92191250 Coffee, dry, acid neutralized
	92191400 Coffee, instant, pre-sweetened with sugar, not reconstituted
	92192000 Coffee, mocha, instant, pre-lightened and pre-sweetened with sugar, not reconstituted
	92192040 Coffee, mocha, instant, decaffeinated, pre-lightened and pre-sweetened with low calorie sweetener, not reconstituted
	92193000 Coffee, instant, pre-lightened and pre-sweetened with sugar, not reconstituted
	92193005 Coffee, instant, decaffeinated, pre-lightened and pre-sweetened with sugar, not reconstituted
	92193020 Coffee, instant, pre-lightened and pre-sweetened with low calorie sweetener, not reconstituted
**Dairy-Based Drinks**	11115400 Kefir, NS as to fat content
	11436000 Yogurt, liquid
	11511000 Chocolate milk, NFS
	11511100 Chocolate milk, ready to drink, whole
	11511200 Chocolate milk, ready to drink, reduced fat
	11511300 Chocolate milk, ready to drink, fat free
	11511400 Chocolate milk, ready to drink, low fat
	11511550 Chocolate milk, ready to drink, reduced sugar, NS as to milk
	11511600 Chocolate milk, ready to drink, low fat (Nesquik)
	11511610 Chocolate milk, ready to drink, fat free (Nesquik)
	11511700 Chocolate milk, ready to drink, low fat, no sugar added (Nesquik)
	11512000 Cocoa, hot chocolate, not from dry mix, made with whole milk
	11512010 Hot chocolate / Cocoa, ready to drink
	11512020 Hot chocolate / Cocoa, ready to drink, made with nonfat milk
	11512100 Hot chocolate / Cocoa, ready to drink, with whipped cream
	11512110 Hot chocolate / Cocoa, ready to drink, made with nonfat milk and whipped cream
	11512500 Hot chocolate, Puerto Rican style, made with whole milk
	11512510 Hot chocolate, Puerto Rican style, made with low fat milk
	11513000 Chocolate milk, made from dry mix, NS as to type of milk
	11513100 Chocolate milk, made from dry mix with whole milk
	11513150 Chocolate milk, made from dry mix with reduced fat milk
	11513200 Chocolate milk, made from dry mix with low fat milk
	11513300 Chocolate milk, made from dry mix with fat free milk
	11513355 Chocolate milk, made from reduced sugar mix with whole milk
	11513360 Chocolate milk, made from reduced sugar mix with reduced fat milk

	11513365 Chocolate milk, made from reduced sugar mix with low fat milk
	11513380 Chocolate milk, made from dry mix, NS as to type of milk (Nesquik)
	11513381 Chocolate milk, made from dry mix with whole milk (Nesquik)
	11513382 Chocolate milk, made from dry mix with reduced fat milk (Nesquik)
	11513383 Chocolate milk, made from dry mix with low fat milk (Nesquik)
	11513384 Chocolate milk, made from dry mix with fat free milk (Nesquik)
	11513391 Chocolate milk, made from no sugar added dry mix with whole milk (Nesquik)
	11513392 Chocolate milk, made from no sugar added dry mix with reduced fat milk (Nesquik)
	11513393 Chocolate milk, made from no sugar added dry mix with low fat milk (Nesquik)
	11513394 Chocolate milk, made from no sugar added dry mix with fat free milk (Nesquik)
	11513400 Chocolate milk, made from syrup, NS as to type of milk
	11513500 Chocolate milk, made from syrup with whole milk
	11513550 Chocolate milk, made from syrup with reduced fat milk
	11513600 Chocolate milk, made from syrup with low fat milk
	11513700 Chocolate milk, made from syrup with fat free milk
	11513801 Chocolate milk, made from light syrup with whole milk
	11513802 Chocolate milk, made from light syrup with reduced fat milk
	11513803 Chocolate milk, made from light syrup with low fat milk
	11513804 Chocolate milk, made from light syrup with fat free milk
	11513851 Chocolate milk, made from sugar free syrup with whole milk
	11513853 Chocolate milk, made from sugar free syrup with low fat milk
	11514100 Hot chocolate / Cocoa, made with dry mix and water
	11514110 Hot chocolate / Cocoa, made with dry mix and whole milk
	11514120 Hot chocolate / Cocoa, made with dry mix and reduced fat milk
	11514130 Hot chocolate / Cocoa, made with dry mix and low fat milk
	11514140 Hot chocolate / Cocoa, made with dry mix and fat free milk
	11514300 Cocoa with nonfat dry milk and low calorie sweetener, mixture, water added
	11514310 Hot chocolate / Cocoa, made with no sugar added dry mix and water
	11514320 Hot chocolate / Cocoa, made with no sugar added dry mix and whole milk
	11514330 Hot chocolate / Cocoa, made with no sugar added dry mix and reduced fat milk
	11514340 Hot chocolate / Cocoa, made with no sugar added dry mix and low fat milk
	11514350 Hot chocolate / Cocoa, made with no sugar added dry mix and fat free milk
	11514500 Cocoa, whey, and low calorie sweetener, mixture, fortified, water added
	11516000 Cocoa, whey, and low-calorie sweetener mixture, low fat milk added
	11518000 Milk beverage with nonfat dry milk and low calorie sweetener, water added, chocolate
	11519000 Milk beverage, made with whole milk, flavors other than chocolate

	11519040 Strawberry milk, NFS
	11519050 Strawberry milk, whole
	11519105 Strawberry milk, reduced fat
	11519200 Strawberry milk, low fat
	11519205 Strawberry milk, fat free
	11519210 Strawberry milk, reduced sugar
	11520000 Milk, malted, unfortified, NS as to flavor, made with milk
	11521000 Milk, malted, unfortified, chocolate, made with milk
	11522000 Milk, malted, unfortified, natural flavor, made with milk
	11525000 Milk, malted, natural flavor, made with milk
	11526000 Milk, malted
	11527000 Milk, malted, fortified, NS as to flavor, made with milk
	11531000 Eggnog, regular
	11531500 Eggnog, low fat / light
	11543000 Milk shake, bottled, chocolate
	11543010 Milk shake, bottled, flavors other than chocolate
	11551050 Licuado or Batido
	11553000 Fruit smoothie drink, made with fruit or fruit juice and dairy products
	11553100 Fruit smoothie, NFS
	11553110 Fruit smoothie, with whole fruit and dairy
	11553120 Fruit smoothie, with whole fruit and dairy, added protein
	11553130 Fruit smoothie juice drink, with dairy
	11560000 Chocolate milk drink
	11560020 Flavored milk drink, whey- and milk-based, flavors other than chocolate
	64134100 Fruit smoothie, light
	78101110 Fruit and vegetable smoothie, added protein
**Energy Drinks**	92650000 Red Bull Energy Drink
	92650005 Red Bull Energy Drink, sugar-free
	92650100 Full Throttle Energy Drink
	92650200 Monster Energy Drink
	92650205 Mountain Dew AMP Energy Drink
	92650210 Mountain Dew AMP Energy Drink, sugar-free
	92650700 Rockstar Energy Drink
	92650705 Rockstar Energy Drink, sugar-free
	92650800 Vault Energy Drink
	92650805 Vault Zero Energy drink
	92651000 Energy drink
	95310200 Energy drink (Full Throttle)
	95310400 Energy drink (Monster)
	95310500 Energy drink (Mountain Dew AMP)
	95310560 Energy drink (NOS)
	95310600 Energy drink (Red Bull)

	95310700 Energy drink (Rockstar)
	95310750 Energy drink (SoBe Energize Energy Juice Drink)
	95310800 Energy drink (Vault)
	95311000 Energy Drink
	95312400 Energy drink, low calorie (Monster)
	95312410 Energy drink, sugar free (Monster)
	95312500 Energy drink, sugar free (Mountain Dew AMP)
	95312550 Energy drink, sugar free (No Fear)
	95312560 Energy drink (Ocean Spray Cran-Energy Juice Drink)
	95312600 Energy drink, sugar-free (Red Bull)
	95312700 Energy drink, sugar free (Rockstar)
	95312800 Energy drink, sugar free (Vault)
	95312900 Energy drink (XS)
	95313200 Energy drink, sugar free
**Flavored Water**	92410110 Carbonated water, sweetened
	92410250 Carbonated water, sweetened, with low-calorie or no-calorie sweetener
	94100200 Water, bottled, sweetened, with low calorie sweetener
	94100300 Water, bottled, flavored (Capri Sun Roarin' Waters)
	94210100 Water, bottled, flavored (Propel Water)
	94210200 Water, bottled, flavored (Glaceau Vitamin Water)
	94210300 Water, bottled, flavored (SoBe Life Water)
	94220100 Propel Zero Water
	94220200 Glaceau Water, low calorie
	94220215 Water, bottled, flavored, sugar free (Glaceau Vitamin Water)
	94220310 Water, bottled, flavored, sugar free (SoBe)
**Nutritional Beverages**	11611000 Instant breakfast, fluid, canned
	11612000 Instant breakfast, powder, milk added
	11613000 Instant breakfast, powder, sweetened with low calorie sweetener, milk added
	11623000 Meal supplement or replacement, commercially prepared, ready-to-drink
	11631000 High calorie beverage, canned or powdered, reconstituted
	11641000 Meal supplement or replacement, milk-based, high protein, liquid
	11641020 Meal replacement or supplement, milk based, ready-to-drink
	11830800 Instant breakfast, powder, not reconstituted
	11830810 Instant breakfast, powder, sweetened with low calorie sweetener, not reconstituted
	11830900 Protein supplement, milk-based, powdered, not reconstituted
	11830940 Meal replacement, high protein, milk based, fruit juice mixable formula, powdered, not reconstituted
	11830970 Meal replacement, protein type, milk-based, powdered, not reconstituted
	11830990 Nutrient supplement, milk-based, powdered, not reconstituted
	11831500 Nutrient supplement, milk-based, high protein, powdered, not reconstituted

	11832000 Meal replacement, protein type, milk- and soy-based, powdered, not reconstituted
	11835100 Meal replacement, Amway's Nutrilite brand Positrim Drink Mix, powdered nonfat dry milk-based, dry, not reconstituted
	11836000 Protein supplement, milk-based, Muscle Milk, powdered, not reconstituted
	11836100 Protein supplement, milk-based, Muscle Milk Light, powdered, not reconstituted
	41430000 Protein powder, NFS
	41430010 Protein supplement, powdered
	41430200 Meal replacement or supplement, soy- and milk-base, powder, reconstituted with water
	41430310 Protein diet powder with soy and casein
	41436000 Nutritional supplement for people with diabetes, liquid
	41440010 Ensure liquid nutrition
	41440020 Ensure with fiber, liquid
	41440050 Ensure Plus liquid nutrition
	41440100 Meal replacement or supplement, liquid, soy-based
	95101000 Nutritional drink or shake, ready-to-drink (Boost)
	95101010 Nutritional drink or shake, ready-to-drink (Boost Plus)
	95102000 Nutritional drink or shake, ready-to-drink (Carnation Instant Breakfast)
	95103000 Nutritional drink or shake, ready-to-drink (Ensure)
	95103010 Nutritional drink or shake, ready-to-drink (Ensure Plus)
	95104000 Nutritional drink or shake, ready-to-drink, sugar free (Glucerna)
	95105000 Nutritional drink or shake, ready-to-drink (Kellogg's Special K Protein)
	95106000 Nutritional drink or shake, ready-to-drink (Muscle Milk)
	95106010 Nutritional drink or shake, ready-to-drink, light (Muscle Milk)
	95110000 Nutritional drink or shake, ready-to-drink (Slim Fast)
	95110010 Nutritional drink or shake, ready-to-drink, sugar free (Slim Fast)
	95110020 Nutritional drink or shake, high protein, ready-to-drink (Slim Fast)
	95120000 Nutritional drink or shake, ready-to-drink, NFS
	95120010 Nutritional drink or shake, high protein, ready-to-drink, NFS
	95120020 Nutritional drink or shake, high protein, light, ready-to-drink, NFS
	95120050 Nutritional drink or shake, liquid, soy-based
	95201000 Nutritional powder mix (Carnation Instant Breakfast)
	95201010 Nutritional powder mix, sugar free (Carnation Instant Breakfast)
	95201200 Nutritional powder mix (EAS Whey Protein Powder)
	95201300 Nutritional powder mix (EAS Soy Protein Powder)
	95201500 Nutritional powder mix, high protein (Herbalife)
	95201600 Nutritional powder mix (Isopure)
	95201700 Nutritional powder mix (Kellogg's Special K20 Protein Water)
	95202000 Nutritional powder mix (Muscle Milk)
	95202010 Nutritional powder mix, light (Muscle Milk)
	95210000 Nutritional powder mix (Slim Fast)

	95210020 Nutritional powder mix, high protein (Slim Fast)
	95220000 Nutritional powder mix, NFS
	95220010 Nutritional powder mix, high protein, NFS
	95230000 Nutritional powder mix, whey based, NFS
	95230010 Nutritional powder mix, protein, soy based, NFS
	95230020 Nutritional powder mix, protein, light, NFS
	95230030 Nutritional powder mix, protein, NFS
**Plain Milk consumed as a beverage**	11100000 Milk, NFS
	11111000 Milk, whole
	11111100 Milk, low sodium, whole
	11111150 Milk, calcium fortified, whole
	11111160 Milk, calcium fortified, low fat (1%)
	11111170 Milk, calcium fortified, fat free (skim)
	11112000 Milk, cow's, fluid, other than whole, NS as to 2%, 1%, or skim (formerly milk, cow's, fluid, "low fat", NS as to percent fat)
	11112110 Milk, reduced fat (2%)
	11112120 Milk, acidophilus, low fat (1%)
	11112130 Milk, acidophilus, reduced fat (2%)
	11112210 Milk, low fat (1%)
	11113000 Milk, fat free (skim)
	11114300 Milk, lactose free, low fat (1%)
	11114320 Milk, lactose free, fat free (skim)
	11114330 Milk, lactose free, reduced fat (2%)
	11114350 Milk, lactose free, whole
	11116000 Goat's milk, whole
**Plant-Based Drinks**	11310000 Milk, imitation, fluid, soy based
	11320000 Soy milk
	11320100 Soy milk, light
	11320200 Soy milk, nonfat
	11321000 Soy milk, chocolate
	11321100 Soy milk, light, chocolate
	11321200 Soy milk, nonfat, chocolate
	11330000 Milk, soy, dry, reconstituted, not baby's
	11340000 Imitation milk, non-soy, sweetened
	11350000 Almond milk, sweetened
	11350010 Almond milk, sweetened, chocolate
	11350020 Almond milk, unsweetened
	11350030 Almond milk, unsweetened, chocolate
	11360000 Rice milk
	11370000 Coconut milk
	11512030 Hot chocolate / Cocoa, ready to drink, made with non-dairy milk

	11512120 Hot chocolate / Cocoa, ready to drink, made with non-dairy milk and whipped cream
	11513310 Chocolate milk, made from dry mix with non-dairy milk
	11513395 Chocolate milk, made from no sugar added dry mix with non-dairy milk (Nesquik)
	11513750 Chocolate milk, made from syrup with non-dairy milk
	11513805 Chocolate milk, made from light syrup with non-dairy milk
	11514150 Hot chocolate / Cocoa, made with dry mix and non-dairy milk
	11514360 Hot chocolate / Cocoa, made with no sugar added dry mix and non-dairy milk
	11519215 Strawberry milk, non-dairy
	92205000 Rice beverage
**Plant water**	42403010 Coconut water, unsweetened
	42404010 Coconut water, sweetened
**Sports Drinks**	92553000 Fruit-flavored thirst quencher beverage, low calorie
	92560000 Fruit-flavored thirst quencher beverage
	92560100 Gatorade Thirst Quencher sports drink
	92560200 Powerade sports drink
	92565000 Fruit-flavored sports drink or thirst quencher beverage, low calorie
	92565100 Gatorade G2 thirst quencher sports drink, low calorie
	92565200 Powerade Zero sports drink, low calorie
	95320200 Sports drink (Gatorade G)
	95320500 Sports drink (Powerade)
	95321000 Sports drink, NFS
	95322200 Sports drink, low calorie (Gatorade G2)
	95322500 Sports drink, low calorie (Powerade Zero)
	95323000 Sports drink, low calorie
**Still Juices and Fruit-Flavored Drinks**	28401200 Gelatin drink, powder, flavored, with low-calorie sweetener, reconstituted
	61201010 Grapefruit juice, 100%, freshly squeezed
	61201020 Grapefruit juice, 100%, NS as to form
	61201220 Grapefruit juice, 100%, canned, bottled or in a carton
	61201225 Grapefruit juice, 100%, with calcium added
	61201620 Grapefruit juice,100%, frozen, reconstituted
	61210000 Orange juice, 100%, NFS
	61210010 Orange juice, 100%, freshly squeezed
	61210220 Orange juice, 100%, canned, bottled or in a carton
	61210230 Orange juice, canned, bottled or in a carton, with sugar
	61210250 Orange juice, 100%, with calcium added, canned, bottled or in a carton
	61210620 Orange juice, 100%, frozen, reconstituted
	61210630 Orange juice, frozen, with sugar (reconstituted with water)
	61210820 Orange juice, 100%, with calcium added, frozen, reconstituted
	61213000 Tangerine juice, NFS
	61213220 Tangerine juice, 100%

	61213620 Tangerine juice, frozen (reconstituted with water)
	61213800 Fruit juice blend, citrus, 100% juice
	61213900 Fruit juice blend, citrus, 100% juice, with calcium added
	64100100 Fruit juice, NFS
	64100110 Fruit juice blend, 100% juice
	64100200 Cranberry juice blend, 100% juice
	64100220 Cranberry juice blend, 100% juice, with calcium added
	64101010 Apple cider
	64104010 Apple juice, 100%
	64104030 Apple juice, 100%, with calcium added
	64104600 Blackberry juice, 100%
	64104610 Blueberry juice
	64105400 Cranberry juice, 100%, not a blend
	64116020 Grape juice, 100%
	64116060 Grape juice, 100%, with calcium added
	64120010 Papaya juice, 100%
	64121000 Passion fruit juice, 100%
	64124020 Pineapple juice, 100%
	64126000 Pomegranate juice, 100%
	64132010 Prune juice, 100%
	64132500 Strawberry juice, 100%
	64133100 Watermelon juice, 100%
	64134000 Fruit smoothie drink, made with fruit or fruit juice only (no dairy products)
	64134015 Fruit smoothie, with whole fruit, no dairy
	64134020 Fruit smoothie, with whole fruit, no dairy, added protein
	64134025 Fruit smoothie, with whole fruit, non-dairy
	64134030 Fruit smoothie juice drink, no dairy
	64134200 Fruit smoothie, bottled
	64200100 Fruit nectar, NFS
	64201010 Apricot nectar
	64201500 Banana nectar
	64202010 Cantaloupe nectar
	64203020 Guava nectar
	64204010 Mango nectar
	64205010 Peach nectar
	64210010 Papaya nectar
	64213010 Passion fruit nectar
	64215010 Pear nectar
	64221010 Soursop, nectar
	73105000 Beet juice
	73105010 Carrot juice, 100%
	74301100 Tomato juice, 100%

	74301150 Tomato juice, 100%, low sodium
	74302000 Tomato juice cocktail
	74303000 Tomato and vegetable juice, 100%
	74303100 Tomato and vegetable juice, 100%, low sodium
	74304000 Tomato juice with clam or beef juice
	75132000 Mixed vegetable juice
	75132100 Celery juice
	75200700 Aloe vera juice drink
	78101000 Vegetable and fruit juice, 100% juice, with high vitamin C
	78101100 Fruit and vegetable smoothie
	78101115 Fruit and vegetable smoothie, non-dairy
	78101118 Fruit and vegetable smoothie, non-dairy, added protein
	78101120 Fruit and vegetable smoothie, bottled
	78101125 Fruit and vegetable smoothie, no dairy
	78101130 Vegetable smoothie
	92510120 Apple-cherry drink
	92510150 Apple juice drink
	92510170 Apple-cranberry-grape juice drink
	92510200 Apple-orange-pineapple juice drink
	92510310 Banana-orange drink
	92510410 Black cherry drink
	92510610 Fruit juice drink
	92510630 Fruit juice drink, NFS
	92510650 Tamarind drink
	92510810 Grapeade and grape drink
	92510820 Grape juice drink
	92510910 Grapefruit juice drink
	92510950 Guava juice drink
	92510955 Lemonade, fruit juice drink
	92510960 Lemonade, fruit-flavored drink
	92511010 Fruit-flavored drink (formerly lemonade)
	92511015 Fruit-flavored drink
	92511020 Lemon-limeade
	92511110 Limeade
	92511190 Orange Juice Drink
	92511200 Orange-mango juice drink
	92511220 Orange drink
	92511240 Orange-lemon drink
	92511250 Fruit juice beverage, 40-50% juice, citrus
	92511260 Orange-cranberry juice drink
	92511270 Orange-peach juice drink
	92511280 Orange-grape-banana juice drink

	92511290 Papaya juice drink
	92511310 Pineapple-grapefruit juice drink
	92511340 Pineapple-orange juice drink
	92511350 Orange-raspberry juice drink
	92511400 Raspberry-flavored drink
	92511510 Strawberry-flavored drink
	92512050 Frozen daiquiri mix, from frozen concentrate, reconstituted
	92513000 Fruit-flavored smoothie drink, frozen, no dairy
	92513010 Fruit-flavored smoothie drink, frozen, light, no dairy
	92520410 Fruit drink, low calorie
	92520810 Grape drink, low calorie
	92520910 Lemonade, low calorie
	92530310 Cherry drink with vitamin C added
	92530410 Fruit-flavored drink, with high vitamin C
	92530510 Cranberry juice drink, with high vitamin C
	92530520 Cranberry-apple juice drink with vitamin C added
	92530610 Fruit juice drink, with high vitamin C
	92530710 Grape drink with vitamin C added
	92530810 Grapefruit juice drink with vitamin C added
	92530840 Guava juice drink with vitamin C added
	92530910 Lemonade with vitamin C added
	92530950 Vegetable and fruit juice drink, with high vitamin C
	92531010 Orange drink and orangeade with vitamin C added
	92531020 Orange breakfast drink, made from frozen concentrate
	92531030 Fruit juice drink (Sunny D)
	92531120 Pineapple-orange juice drink with vitamin C added
	92531210 Strawberry-flavored drink with vitamin C added
	92541010 Fruit-flavored drink, powdered, reconstituted
	92541020 Lemonade-flavored drink, made from powdered mix, with sugar
	92541040 Lemonade-flavored drink, made from powdered mix, low calorie
	92541100 Apple cider-flavored drink, made from powdered mix, with sugar and vitamin C added
	92542000 Fruit-flavored drink, with high vitamin C, powdered, reconstituted
	92544000 Fruit-flavored drink, made from unsweetened powdered mix (fortified with vitamin C), with sugar added in preparation
	92550030 Fruit juice drink, with high vitamin C, light
	92550035 Fruit juice drink, light
	92550040 Fruit juice drink, diet
	92550050 Apple-white grape juice drink, low calorie, with vitamin C added
	92550110 Cranberry juice drink, with high vitamin C, light
	92550200 Grape juice drink, light
	92550210 Cranberry-apple juice drink, low calorie, with vitamin C added

	92550300 Grapefruit juice drink, low calorie, with vitamin C added
	92550350 Orange juice beverage, 40-50% juice, light
	92550360 Apple juice beverage, 40-50% juice, light
	92550370 Lemonade, fruit juice drink, light
	92550380 Pomegranate juice beverage, 40-50% juice, light
	92550400 Vegetable and fruit juice drink, with high vitamin C, diet
	92550405 Vegetable and fruit juice drink, with high vitamin C, light
	92550610 Fruit-flavored drink, with high vitamin C, diet
	92550620 Fruit-flavored drink, diet
	92551600 Citrus Juice Drink, Low Calorie
	92551700 Juice drink, low calorie
	92552000 Fruit-flavored drink, with high vitamin C, powdered, reconstituted, diet
	92552010 Fruit-flavored drink, powdered, reconstituted, diet
	92552020 Fruit juice drink, reduced sugar (Sunny D)
	92552030 Fruit juice drink (Capri Sun)
	92552050 Orange Breakfast Drink, Low Calorie
	92552100 Orange-cranberry juice drink, low calorie, with vitamin C added
	92582100 Fruit juice drink, with high vitamin C, plus added calcium
	92582110 Fruit juice drink, added calcium (Sunny D)
	92582120 Fruit-flavored drink, reduced sugar, with high vitamin C, plus added calcium
	92731000 Fruit-flavored drink, non-carbonated, made from powdered mix, with sugar
	92741000 Fruit-flavored drink, non-carbonated, made from low calorie powdered mix
	95341000 FUZE Slenderize fortified low calorie fruit juice beverage
	95342000 Fruit juice, acai blend
**Tap water**	94000100 Water, tap
**Tea**	92204000 Mate, sweetened beverage made from dried green leaves
	92301000 Tea, NS as to type, unsweetened
	92301060 Tea, NS as to type, presweetened with sugar
	92301080 Tea, NS as to type, presweetened with low calorie sweetener
	92301100 Tea, NS as to type, decaffeinated, unsweetened
	92301130 Tea, NS as to type, presweetened, NS as to sweetener
	92301160 Tea, NS as to type, decaffeinated, presweetened with sugar
	92301180 Tea, NS as to type, decaffeinated, presweetened with low calorie sweetener
	92301190 Tea, NS as to type, decaffeinated, presweetened, NS as to sweetener
	92302000 Tea, hot, leaf, black
	92302200 Tea, leaf, presweetened with sugar
	92302300 Tea, leaf, presweetened with low calorie sweetener
	92302400 Tea, leaf, presweetened, NS as to sweetener
	92302500 Tea, hot, leaf, black, decaffeinated
	92302600 Tea, leaf, decaffeinated, presweetened with sugar
	92302700 Tea, leaf, decaffeinated, presweetened with low calorie sweetener

	92302800 Tea, leaf, decaffeinated, presweetened, NS as to sweetener
	92303010 Tea, hot, leaf, green
	92303100 Tea, hot, leaf, green, decaffeinated
	92304000 Tea, made from frozen concentrate, unsweetened
	92304100 Tea, hot, leaf, oolong
	92305000 Tea, made from powdered instant, presweetened, NS as to sweetener
	92305010 Tea, iced, instant, black, unsweetened
	92305040 Tea, iced, instant, black, pre-sweetened with sugar
	92305050 Tea, iced, instant, black, decaffeinated, pre-sweetened with sugar
	92305090 Tea, iced, instant, black, pre-sweetened with low calorie sweetener
	92305110 Tea, iced, instant, black, decaffeinated, pre-sweetened with low calorie sweetener
	92305180 Tea, iced, instant, black, decaffeinated, unsweetened
	92305800 Tea, made from powdered instant, decaffeinated, presweetened, NS as to sweetener
	92305900 Tea, iced, instant, green, unsweetened
	92305910 Tea, iced, instant, green, pre-sweetened with sugar
	92305920 Tea, iced, instant, green, pre-sweetened with low calorie sweetener
	92306000 Tea, hot, herbal
	92306020 Tea, herbal, presweetened with sugar
	92306030 Tea, herbal, presweetened with low calorie sweetener
	92306040 Tea, herbal, presweetened, NS as to sweetener
	92306090 Tea, hot, hibiscus
	92306700 Tea, hot, chamomile
	92306800 Tea, hot, chai, with milk
	92307000 Tea, iced, instant, black, unsweetened, dry
	92307400 Tea, iced, instant, black, pre-sweetened, dry
	92307500 Iced Tea / Lemonade juice drink
	92307510 Iced Tea / Lemonade juice drink, light
	92307520 Iced Tea / Lemonade juice drink, diet
	92308000 Tea, iced, brewed, black, pre-sweetened with sugar
	92308010 Tea, iced, brewed, black, pre-sweetened with low calorie sweetener
	92308020 Tea, iced, brewed, black, unsweetened
	92308030 Tea, iced, brewed, black, decaffeinated, pre-sweetened with sugar
	92308040 Tea, iced, brewed, black, decaffeinated, pre-sweetened with low calorie sweetener
	92308050 Tea, iced, brewed, black, decaffeinated, unsweetened
	92308500 Tea, iced, brewed, green, pre-sweetened with sugar
	92308510 Tea, iced, brewed, green, pre-sweetened with low calorie sweetener
	92308520 Tea, iced, brewed, green, unsweetened
	92308530 Tea, iced, brewed, green, decaffeinated, pre-sweetened with sugar
	92308540 Tea, iced, brewed, green, decaffeinated, pre-sweetened with low calorie sweetener

	92308550 Tea, iced, brewed, green, decaffeinated, unsweetened
	92309000 Tea, iced, bottled, black
	92309010 Tea, iced, bottled, black, decaffeinated
	92309020 Tea, iced, bottled, black, diet
	92309030 Tea, iced, bottled, black, decaffeinated, diet
	92309040 Tea, iced, bottled, black, unsweetened
	92309050 Tea, iced, bottled, black, decaffeinated, unsweetened
	92309500 Tea, iced, bottled, green
	92309510 Tea, iced, bottled, green, diet
	92309520 Tea, iced, bottled, green, unsweetened

Note: Codes in powdered form were converted to reconstituted/prepared amount.

Supplemental Table S2. Consumers and daily intakes (mL) for all beverage types combined and by beverage type among subgroups 13-19 y, 20-49 y, and 50-64 y

		**13-19 y**		**20-49 y**		**50-64 y**	
**Beverage Type**	**Statistics (mL/day)**	**ABA-BSW 2021** (n=2335)	**NHANES 2017-2018** (n=916)	**ABA-BSW 2021** (n=13475)	**NHANES 2017-2018** (n=2136)	**ABA-BSW 2021** (n=4743)	**NHANES 2017-2018** (n=1384)
All beverages (excluding tap water)	% Consumers	97.9%	94.2%	99.0%	96.5%	98.5%	99.1%
	Per Capita Mean	1576	1247	1972	1703	1822	1741
	Per Consumer Estimates						
	Mean	1610	1324	1993	1765	1850	1757
	P10	375	326	472	490	547	503
	P25	725	594	899	870	975	869
	P50	1303	1100	1599	1449	1610	1542
	P75	2196	1698	2627	2262	2439	2316
	P90	3171	2467	3895	3355	3441	3292
	P90:P10 ratio	8	8	8	7	6	7
Bottled water	% Consumers	62.4%	49.0%	48.2%	51.3%	42.9%	50.6%
	Per Capita Mean	559	577	405	748	415	639
	Per Consumer Estimates						
	Mean	895	1178	842	1457	966	1262
	P10	181	315	159	360	260	360
	P25	361	507	318	507	414	507
	P50	681	887	572	1029	827	1014
	P75	1282	1521	1147	1920	1371	1680
	P90	1972	2195	2008	3030	2068	2535
	P90:P10 ratio	11	7	13	8	8	7
CSD (total)	% Consumers	44.1%	40.8%	56.9%	35.3%	50.3%	34.4%
	Per Capita Mean	233	208	404	247	415	244
	Per Consumer Estimates						
	Mean	528	510	710	699	826	710
	P10	114	161	125	217	186	161

		13-19 y		20-49 y		50-64 y	
**Beverage Type**	**Statistics (mL/day)**	**ABA-BSW 2021** (n=2335)	**NHANES 2017-2018** (n=916)	**ABA-BSW 2021** (n=13475)	**NHANES 2017-2018** (n=2136)	**ABA-BSW 2021** (n=4743)	**NHANES 2017-2018** (n=1384)
	P25	181	326	260	334	315	333
	P50	364	388	520	496	591	496
	P75	675	620	903	899	1072	805
	P90	1182	973	1513	1364	1688	1364
	P90:P10 ratio	10	6	12	6	9	8
Regular CSD	% Consumers	40.0%	37.5%	49.5%	30.8%	32.8%	24.9%
	Per Capita Mean	208	192	335	205	249	161
	Per Consumer Estimates						
	Mean	521	512	677	665	758	646
	P10	114	155	114	217	181	161
	P25	181	287	250	334	296	310
	P50	361	403	472	478	591	403
	P75	666	635	887	805	1005	744
	P90	1144	1000	1478	1364	1608	1240
	P90:P10 ratio	10	6	13	6	9	8
LNCS CSD	% Consumers	6.0%	3.9%	12.4%	5.0%	19.5%	10.3%
	Per Capita Mean	24	16	68	42	167	83
	Per Consumer Estimates						
	Mean	405	409	551	841	857	806
	P10	100	240	110	265	204	240
	P25	181	360	181	360	296	345
	P50	296	360	364	495	591	600
	P75	536	360	682	1080	1126	1080
	P90	832	949	1245	2094	1766	1521
	P90:P10 ratio	8	4	11	8	9	6
Coffee	% Consumers	28.0%	11.6%	56.4%	47.8%	62.4%	62.9%

		13-19 y		20-49 y		50-64 y	
**Beverage Type**	**Statistics (mL/day)**	**ABA-BSW 2021** (n=2335)	**NHANES 2017-2018** (n=916)	**ABA-BSW 2021** (n=13475)	**NHANES 2017-2018** (n=2136)	**ABA-BSW 2021** (n=4743)	**NHANES 2017-2018** (n=1384)
	Per Capita Mean	124	44	259	257	362	374
	Per Consumer Estimates						
	Mean	443	377	459	537	581	594
	P10	125	180	125	180	208	240
	P25	224	255	224	255	281	315
	P50	355	326	355	372	472	450
	P75	556	496	591	620	710	720
	P90	851	543	944	960	1180	1080
	P90:P10 ratio	7	3	8	5	6	5
Dairy-based beverages	% Consumers	15.5%	11.7%	16.9%	3.8%	10.3%	3.8%
	Per Capita Mean	46	39	57	18	32	14
	Per Consumer Estimates						
	Mean	298	335	334	474	317	377
	P10	69	124	74	186	95	189
	P25	125	248	125	248	159	264
	P50	247	248	247	368	260	391
	P75	369	432	414	543	414	473
	P90	591	496	654	899	591	543
	P90:P10 ratio	9	4	9	5	6	3
Energy drinks	% Consumers	14.1%	1.2%	26.8%	4.7%	8.8%	2.4%
	Per Capita Mean	66	7	111	26	40	14
	Per Consumer Estimates						
	Mean	470	577	416	561	456	575
	P10	125	372	112	252	125	120
	P25	260	480	181	372	186	124
	P50	414	496	296	496	414	248
	P75	536	729	536	744	536	620

		**13-19 y**		**20-49 y**		**50-64 y**	
**Beverage Type**	**Statistics (mL/day)**	**ABA-BSW 2021** (n=2335)	**NHANES 2017-2018** (n=916)	**ABA-BSW 2021** (n=13475)	**NHANES 2017-2018** (n=2136)	**ABA-BSW 2021** (n=4743)	**NHANES 2017-2018** (n=1384)
	P90	830	930	827	960	887	1800
	P90:P10 ratio	7	3	7	4	7	15
Flavored water	% Consumers	13.1%	1.4%	18.6%	2.0%	10.1%	2.9%
	Per Capita Mean	70	5	107	14	72	20
	Per Consumer Estimates						
	Mean	536	362	577	705	707	676
	P10	110	180	114	360	181	120
	P25	189	180	235	435	296	495
	P50	414	315	414	600	536	525
	P75	722	507	732	960	1013	960
	P90	1089	720	1214	1080	1486	1521
	P90:P10 ratio	10	4	11	3	8	13
Nutritional beverages	% Consumers	7.4%	3.4%	11.7%	5.5%	6.5%	3.4%
	Per Capita Mean	27	18	44	24	23	13
	Per Consumer Estimates						
	Mean	364	541	375	437	363	371
	P10	79	192	79	217	116	213
	P25	159	240	159	240	181	248
	P50	296	480	296	384	296	336
	P75	414	640	414	541	414	384
	P90	827	1280	676	720	591	640
	P90:P10 ratio	11	7	9	3	5	3
Plain milk	% Consumers	22.7%	14.3%	25.4%	8.3%	20.8%	11.4%
	Per Capita Mean	84	51	96	34	77	42
	Per Consumer Estimates						
	Mean	372	357	378	404	370	373

		**13-19 y**		**20-49 y**		**50-64 y**	
**Beverage Type**	**Statistics (mL/day)**	**ABA-BSW 2021** (n=2335)	**NHANES 2017-2018** (n=916)	**ABA-BSW 2021** (n=13475)	**NHANES 2017-2018** (n=2136)	**ABA-BSW 2021** (n=4743)	**NHANES 2017-2018** (n=1384)
	P10	74	153	76	153	112	153
	P25	134	244	150	244	181	229
	P50	260	336	261	320	296	336
	P75	410	442	415	488	414	503
	P90	675	534	784	808	720	534
	P90:P10 ratio	9	4	10	5	6	4
Plant-based drinks	% Consumers	5.4%	3.1%	11.5%	4.5%	8.8%	4.8%
	Per Capita Mean	15	8	38	10	28	9
	Per Consumer Estimates						
	Mean	285	272	327	232	324	190
	P10	74	107	63	31	48	61
	P25	114	173	125	107	125	61
	P50	204	320	249	244	208	122
	P75	323	351	414	356	361	259
	P90	572	351	650	397	650	366
	P90:P10 ratio	8	3	10	13	14	6
Plant water	% Consumers	2.8%	0.2%	1.8%	0.7%	1.2%	0.4%
	Per Capita Mean	10	1	8	4	6	2
	Per Consumer Estimates						
	Mean	357	463	440	604	545	525
	P10	63	80	112	300	114	240
	P25	159	480	204	600	261	360
	P50	296	480	406	690	414	525
	P75	536	480	536	690	648	525
	P90	536	630	783	690	1241	720
	P90:P10 ratio	9	8	7	2	11	3
	% Consumers	19.6%	7.9%	23.6%	4.8%	11.9%	3.1%

		**13-19 y**		**20-49 y**		**50-64 y**	
**Beverage Type**	**Statistics (mL/day)**	**ABA-BSW 2021** (n=2335)	**NHANES 2017-2018** (n=916)	**ABA-BSW 2021** (n=13475)	**NHANES 2017-2018** (n=2136)	**ABA-BSW 2021** (n=4743)	**NHANES 2017-2018** (n=1384)
Sports drinks	Per Capita Mean	102	45	118	33	66	22
	Per Consumer Estimates						
	Mean	519	572	499	693	557	714
	P10	125	217	112	310	138	248
	P25	186	372	186	372	296	372
	P50	414	620	410	620	414	744
	P75	650	744	650	930	650	744
	P90	1080	992	1072	992	1072	992
	P90:P10 ratio	9	5	10	3	8	4
Still juices and fruit-flavored drinks	% Consumers	39.6%	32.1%	36.6%	24.1%	26.6%	24.4%
	Per Capita Mean	180	146	177	117	117	107
	Per Consumer Estimates						
	Mean	455	456	482	484	442	437
	P10	114	155	114	155	112	109
	P25	181	233	186	242	181	186
	P50	328	349	338	357	328	345
	P75	556	543	591	543	536	524
	P90	968	899	984	870	960	861
	P90:P10 ratio	9	6	9	6	9	8
Tea	% Consumers	20.3%	19.8%	36.3%	27.3%	32.3%	32.2%
	Per Capita Mean	93	96	170	171	194	241
	Per Consumer Estimates						
	Mean	458	485	467	628	602	748
	P10	125	184	110	195	181	195
	P25	208	248	204	300	236	345
	P50	355	372	355	496	414	599
	P75	536	543	577	761	710	913

		**13-19 y**		**20-49 y**		**50-64 y**	
**Beverage Type**	**Statistics (mL/day)**	**ABA-BSW 2021** (n=2335)	**NHANES 2017-2018** (n=916)	**ABA-BSW 2021** (n=13475)	**NHANES 2017-2018** (n=2136)	**ABA-BSW 2021** (n=4743)	**NHANES 2017-2018** (n=1384)
	P90	934	989	947	1240	1246	1522
	P90:P10 ratio	7	5	9	6	7	8
Tap water [1]	% Consumers	77.4%	47.1%	84.7%	47.6%	77.8%	45.%
	Per Capita Mean	735	465	870	734	784	590
	Per Consumer Estimates						
	Mean	950	988	1027	1542	1008	1312
	P10	250	278	250	315	250	240
	P25	375	435	375	585	375	525
	P50	750	740	750	1200	750	1014
	P75	1250	1275	1250	2067	1250	1890
	P90	1750	2029	1750	3240	1750	2670
	P90:P10 ratio	7	7	7	10	7	11

[1] Estimates for tap water not directly comparable between surveys as NHANES estimates are based on 24-hr dietary recalls while ABA-BSW estimates are based on typical daily amounts from a FFQ (tap water was not an option to select in the online 24-hr dietary recall survey for ABA-BSW%.

Supplemental Table S3A. Beverage COs among ages 13-19 years

	ABA-BSW 2021			NHANES 2017-2018		
	Total	Average		Total	Average	
**Beverage Type**	**Number of COs [1]**	**COs/day**	**mL/CO**	**Number of COs**	**COs/day**	**mL/CO**
All beverages (excluding tap water)	12146	5.4	300	2417	2.8	467
Bottled water	3802	2.7	328	807	1.8	656
CSD (total)	2000	1.9	273	478	1.3	402
Regular CSD	1771	1.9	275	449	1.3	409
LNCS CSD	229	1.6	261	29	1.2	334
Coffee	920	1.4	313	100	1.1	338
Dairy-based drinks	447	1.3	226	108	1.2	288
Energy drinks	439	1.4	344	11	1.0	551
Flavored water	554	1.9	284	14	1.1	340
Nutritional beverages	210	1.4	264	25	1.3	425
Plain milk	691	1.4	261	149	1.2	309
Plant-based drinks	169	1.3	216	27	1.1	258
Plant water	94	1.4	255	4	1.2	383
Sports drinks	604	1.4	364	77	1.1	525
Still juices and fruit-flavored drinks	1472	1.6	278	421	1.3	350
Tea	744	1.5	299	196	1.2	415

[1] Unweighted number of COs; all other statistics are appropriately weighted using survey weights.

Supplemental Table S3B. Beverage COs among ages among ages 20-49 years

	ABA-BSW 2021			NHANES 2017-2018		
	Total	Average		Total	Average	
**Beverage Type**	**Number of COs [1]**	**COs/day**	**mL/CO**	**Number of COs**	**COs/day**	**mL/CO**
All beverages (excluding tap water)	86892	6.5	304	6982	3.3	534
Bottled water	15482	2.4	345	2335	2.0	735
CSD (total)	16960	2.2	325	1213	1.5	473
Regular CSD	14098	2.1	324	1063	1.4	460
LNCS CSD	2862	1.7	326	150	1.5	549
Coffee	12841	1.7	275	1136	1.2	433
Dairy-based drinks	2901	1.3	251	113	1.1	444
Energy drinks	4959	1.4	296	93	1.0	541
Flavored water	4738	1.9	298	38	1.3	532
Nutritional beverages	2153	1.4	269	93	1.2	357
Plain milk	4693	1.4	263	210	1.2	349
Plant-based drinks	1979	1.3	244	101	1.4	166
Plant water	324	1.3	329	16	1.6	368
Sports drinks	4363	1.4	346	121	1.1	638
Still juices and fruit-flavored drinks	7473	1.6	303	761	1.2	396
Tea	8026	1.6	285	752	1.3	481

[1] Unweighted number of COs; all other statistics are appropriately weighted using survey weights.

Supplemental Table S3C. Beverage COs among ages among ages 50-64 years

	**ABA-BSW 2021**			**NHANES 2017-2018**		
	**Total Number**	**Average**		**Total Number**	**Average**	
**Beverage Type**	**of COs [1]**	**COs/day**	**mL/CO**	**of COs**	**COs/day**	**mL/CO**
All beverages (excluding tap water)	25473	5.4	341	4896	3.6	487
Bottled water	5290	2.6	370	1656	2.1	605
CSD (total)	5275	2.2	378	675	1.5	462
Regular CSD	3286	2.1	364	524	1.5	445
LNCS CSD	1989	2.1	400	151	1.6	499
Coffee	5524	1.9	312	1017	1.3	460
Dairy-based drinks	549	1.2	263	54	1.1	355
Energy drinks	517	1.4	332	22	1.2	474
Flavored water	949	2.0	357	36	1.5	447
Nutritional beverages	381	1.3	287	37	1.1	329
Plain milk	1235	1.3	289	150	1.1	333
Plant-based drinks	493	1.2	259	66	1.2	160
Plant water	79	1.7	312	11	1.0	525
Sports drinks	699	1.4	413	52	1.2	607
Still juices and fruit-flavored drinks	1828	1.5	299	492	1.3	338
Tea	2654	1.8	340	628	1.5	500

[1] Unweighted number of COs; all other statistics are appropriately weighted using survey weights.

Xiaoyu Bi -- Beverage consumption patterns among U.S. adolescents and adults from a 24-hour dietary recall survey conducted in 2021 compared to the National Health and Nutrition Examination Survey (NHANES) 2017-2018

S-1A

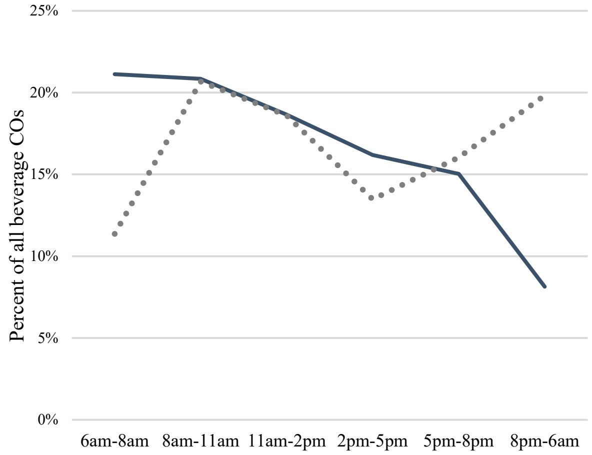

S-1B

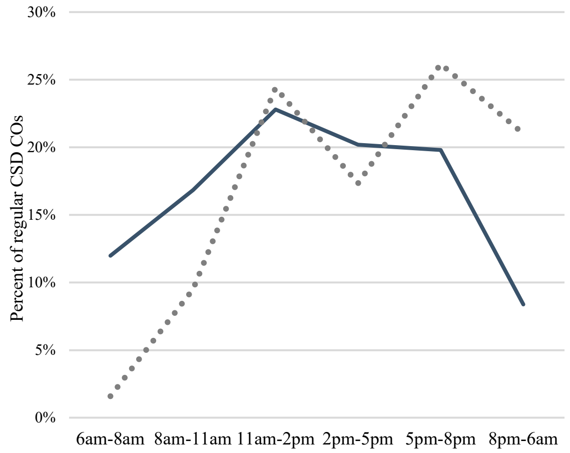

S-1C

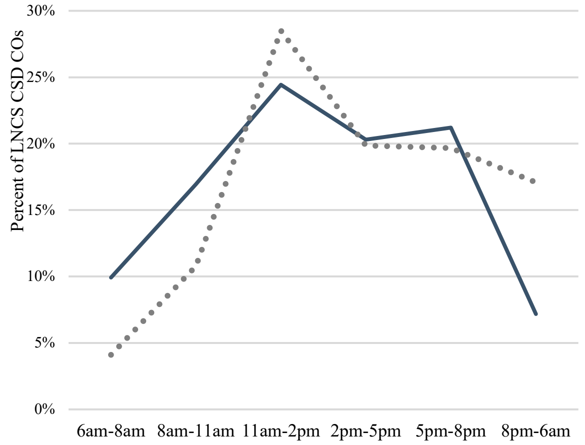

S-1D

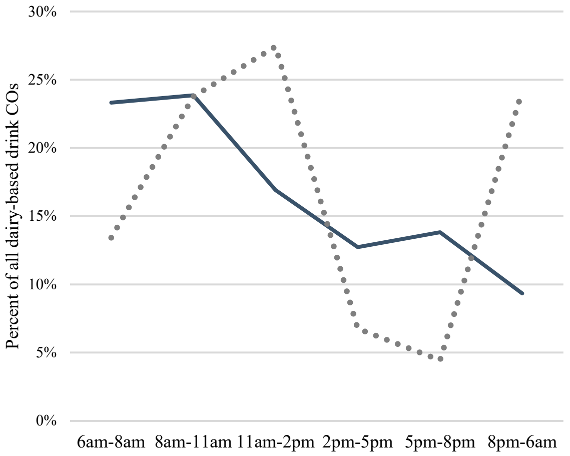

S-1E

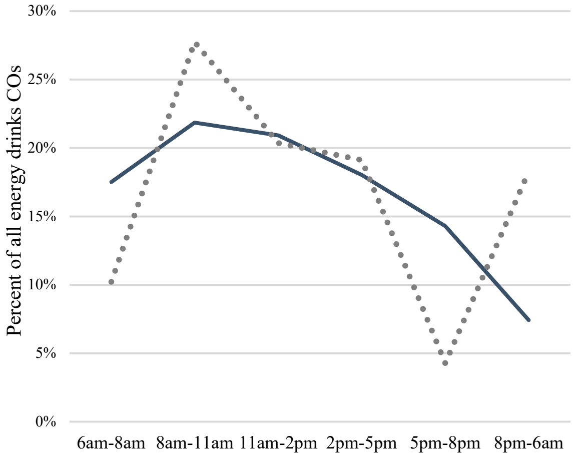

S-1F

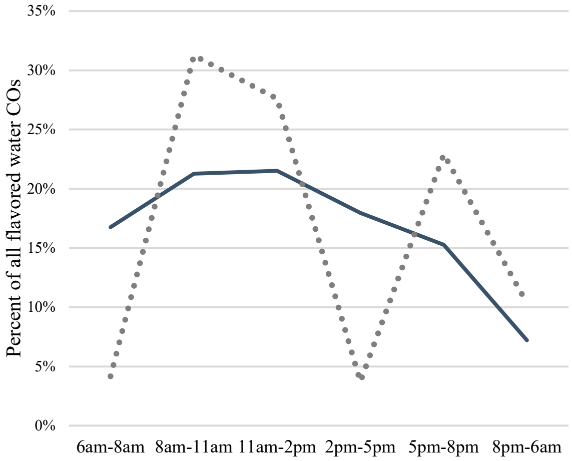

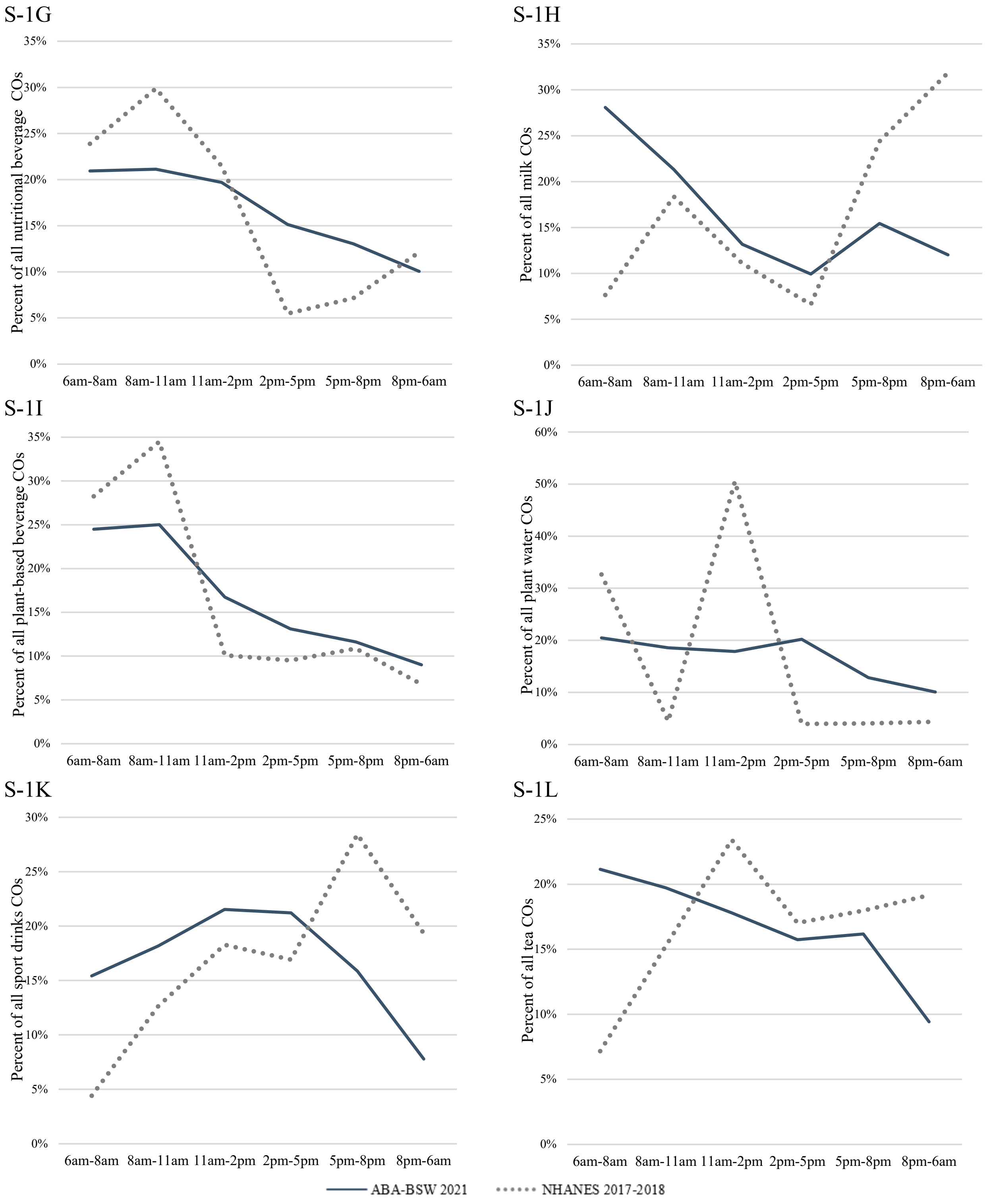

Supplemental Figure S1. Percentage of distribution of beverage COs within six time intervals among consumer ages 13-64 years by survey; (1A) all beverage types combined, (1B) Regular CSD, (1C) LNCS CSD, (1D) dairy-based drinks, (1E) energy drinks, (1F) flavored water, (1G) nutritional beverages, (1H) plain milk, (1I) plant-based drinks, (1J) plant water, (1K) sport drinks, and (1L) tea.
