# Supplementary material for: Beverage Consumption Patterns among U.S. Adolescents and Adults from a New 24-h Beverage Recall Survey Compared to the National Health and Nutrition Examination Survey (NHANES) 2017–2018
Source: Nutrients. 2023 Aug 12;15(16):3561. doi: 10.3390/nu15163561 (PMC10459501; doi:10.3390/nu15163561)
Supplement: Supplementary file 1 [file nutrients-15-03561-s001.zip › Supplemental File -- ABA_BSW Survey Design and Interface.pdf]

Supplemental File: Online format of ABA-BSW survey to participants

Survey Introduction| Opening page

Greetings!
Hello. Thank you for taking time to participate in this study. Brandscapes Worldwide, is a global, independent Market Research company that abides by the ESOMAR code of conduct. We carry out surveys among users of different products and services. We are conducting research on non-alcoholic beverages. Please spare 20 minutes to answer a few questions.

- Your participation in this study is completely voluntary. If you feel uncomfortable answering any questions, you can withdraw from the survey at any point.
- Your responses will be strictly confidential and data from this research will be reported only in the aggregate.

PLEASE START THE SURVEY NOW BY CLICKING ON THE CONTINUE BUTTON BELOW.

Continue >

SECTION 1 : Screening and demographics

Screener and Demographics/1

Before we start the survey, we would like to know if you or any other member in your household or any close friend works in any of the following organizations.

Please select as many as apply.

Advertising Agency	Market Research Agency	Journalism	Company manufacturing any type of beverage/s
Company distributing any type of beverage/s	Banks	Government Organization	Other

Are you....?

Please select one only.

- Female
- Male
- Decline to Answer

Please enter your age in years since your last birthday

Please type a number into the box(es) below

30

Presently, are you...?

Please select one only.

- Single
- Married/ In a Live-in Relationship
- Widowed/ Divorced/ Separated
- Decline to Answer

How many people typically live in your household including yourself?

Please select one only.

- 1
- 2
- 3
- 4
- 5
- 6 or more

Which of the following best describes your current employment status or Primary employement status?

Please select one only.

- Employed full-time
- Employed part-time
- Not employed
- Student
- Retired
- Homemaker
- Decline to Answer

All participants between the age of 13- 64 yrs were allowed to take the survey except those except those that worked at an advertising or market research agency or beverage company. The age, sex, marital status, employment status and number of members in household was captured for all participants.

Screener and Demographics/2

Please select your state of current residence?
Please select one only.

Alabama	Alaska	Arizona
Arkansas	California	Colorado
Connecticut	Delaware	District of Columbia
Florida	Georgia	Hawaii
Idaho	Illinois	Indiana
Iowa	Kansas	Kentucky
Louisiana	Maine	Maryland
Massachusetts	Michigan	Minnesota
Mississippi	Missouri	Montana
Nebraska	Nevada	New Hampshire
New Jersey	New Mexico	New York
North Carolina	North Dakota	Ohio
Oklahoma	Oregon	Pennsylvania
Rhode Island	South Carolina	South Dakota
Tennessee	Texas	Utah
Vermont	Virginia	Washington
West Virginia	Wisconsin	Wyoming

Continue >

You live in **Alabama**. Does the area you live in fall under Urban or Rural limits?
Please select one only.

- Urban
- Rural
- Don't Know

Continue >

Please select the ethnicity/ race you belong to?
Please select one only.

- Non-Hispanic white
- Non-Hispanic black
- Hispanic
- Asian
- Other/Mixed
- Don't Know
- Decline to Answer

Continue >

Please think about the average annual income of all the members of your household from all sources (salary,wages, pensions, scholarships) In which of these groups would you place your household?
Please select one only.

- Less than $24,000
- $24,000- $50,000
- $51,000 - $90,000
- More than $90,000
- Decline to Answer

Continue >

We also recorded state of current residence, region (urban, rural), household income, ethnicity, weight, and height across participants.

SECTION 2 : 24-hour beverage recall

Incidence, frequency, and time of consumption of beverages in 24 hrs.

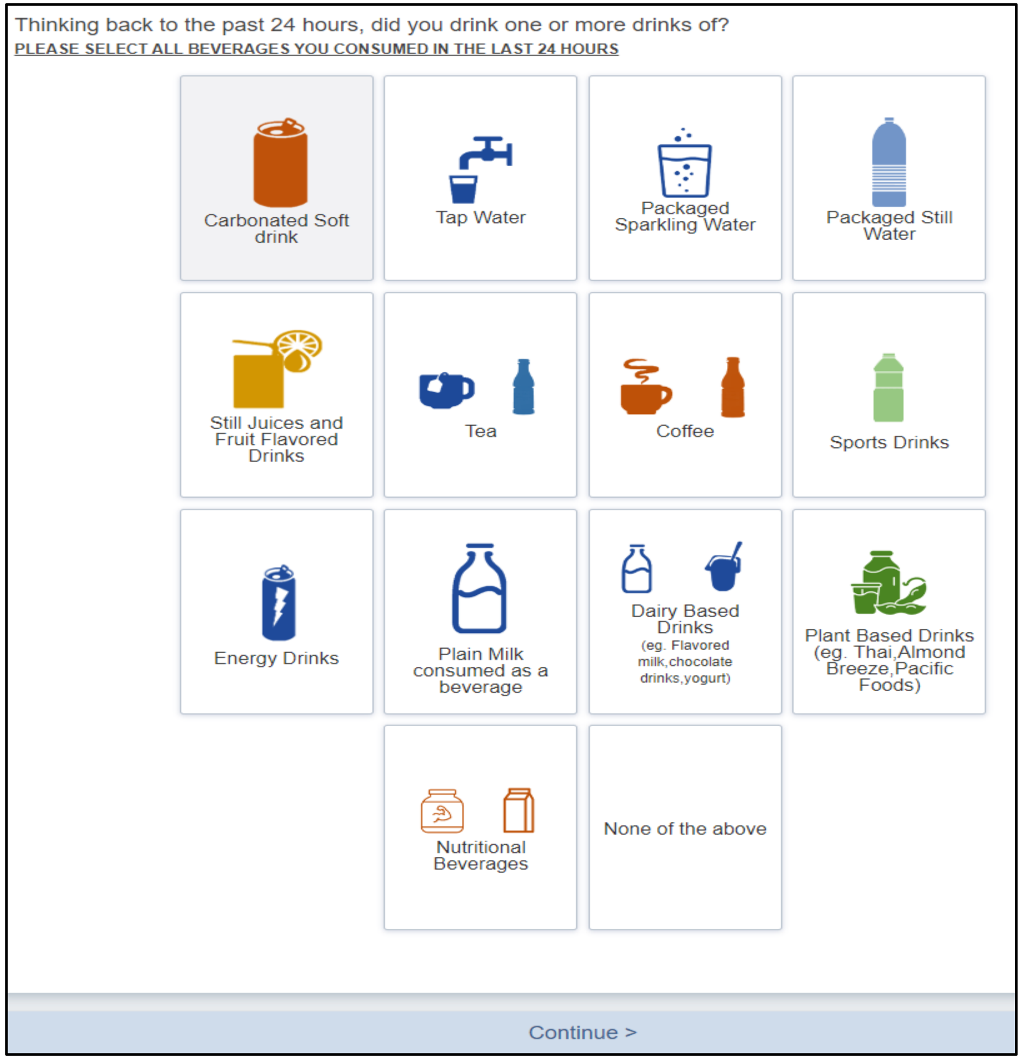

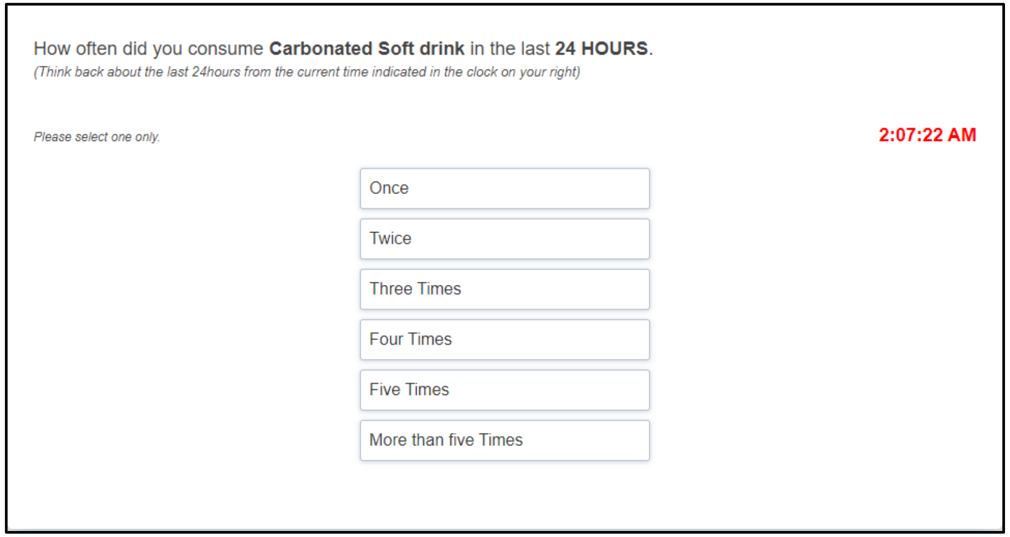

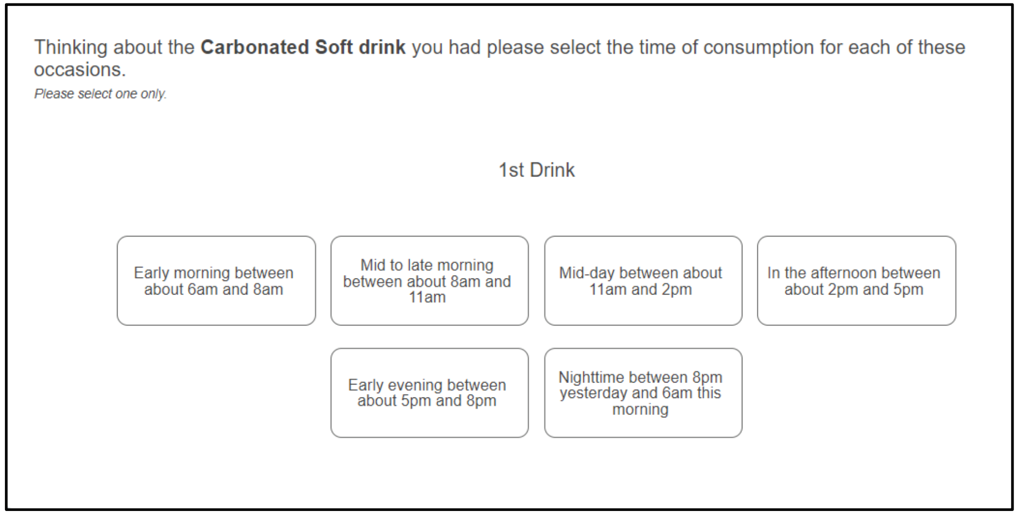

All participants had to record all beverages consumed in the past 24 hours, their frequency of consumption and the time of consumption for each consumption occasion. Participants were allowed to select from 13 different beverage types.

Beverage brand and type (caff. /decaff. and regular/diet) and place

Please select the brand of beverage of **Carbonated Soft drink**, for your **1st drink Early morning between about 6am and 8am**.

Please select one only.

Coca-Cola	Pepsi-Cola	Dr Pepper	Sprite
Mountain Dew	Fanta	Canada Dry	7-Up
Sierra Mist	Barqs	A&W	Mug

Others (Pls specify)

Please select the type of beverage of **"Coca-Cola"** which you had.

Please select one only.

**Caffeinated**	
Regular	○
Diet	●
**Decaffeinated**	
Regular	○
Diet	○

Please select **where you** consumed it?

Please select one only.

At Home

Away from Home (consumed on the go, at work, in a restaurant/cafe)

The survey asked the participants to indicate the brand of the beverage and, if relevant, whether the brand they consumed was caffeinated or decaffeinated. The place of consumption at home / away from home was also recorded.

SECTION 2a. Container type, size, and volume for ready-to-drink beverages.

Pack type and size

What was the container from which you drank **Carbonated Soft drink** for your **1st drink Early morning between about 6am and 8am** e.g.,drinking from a glass?

Please select one only.

- Can
- Bottle (Glass, Plastic, Aluminium)
- Fountain Cup
- Jug
- Cup/Glass
- Any other (pls specify)

The pack type and pack size for each occasion of beverage consumption was recorded. This was repeated for each consumption occasion by beverage.

How big was the container that you drank the beverage from?

Please select the size in millilitres / litres / ounce.

- Up to 4 oz(125ml)
- More than 4 oz (125ml) to 8 oz (236ml)
- More than 8 oz (236ml) to 12 oz (355ml)
- More than 12 oz (355 ml) to 16 oz (472ml)
- More than 16 oz (472ml) to 20 oz (600ml)
- More than 20 oz (600ml) to 24 oz (700ml)
- More than 24 oz (700ml) to 32 oz (960ml)
- More than 32 oz (960 ml) to 40 oz (1.2l)
- More than 40 oz(1.2l) to 50 oz (1.5l)
- Any other (pls specify volume with units in ml/oz)

Volume consumed

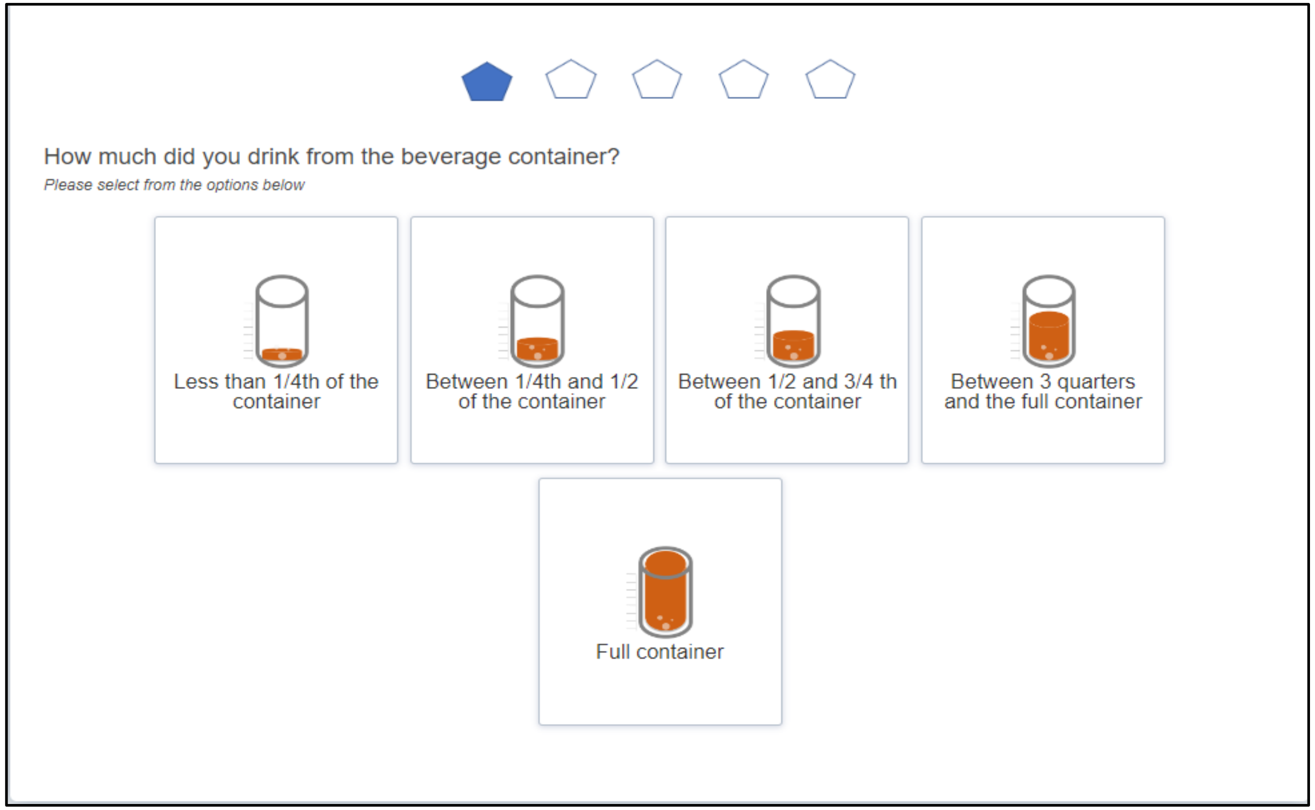

The participants had to record the proportion of beverage consumed within each CO

SECTION 2b. Container type, size, and volume for freshly-prepared beverages.

Type of Beverage (packaged vs freshly prepared) & preparation

Was the Coffee you had for your 1st drink..?

Please select one only.

- Packaged
- Freshly Prepared

You mentioned that you had **Coffee** for **1st drink Mid to late morning between about 8am and 11am**.
From the list below select the format which was used for beverage preparation for each occasion of consumption.

Please select one only.

- Prepared from grounds/beans
- Prepared from PODS
- Any other(Pls specify)

In case of beverages such as coffee, tea and juice, which can be consumed in both packaged and freshly prepared formats, a question was asked to determine the format. In case of freshly prepared the following question were asked. In case of packaged similar route followed as mentioned in Section 2a.

Type of coffee, quantity used to prepare

You mentioned that you had **Coffee** for your **1st drink Mid to late morning between about 8am and 11am**. Select the type of beverage you had from the list below?

Please select one only.

- Cappuccino
- Latte
- Black Coffee /Regular Coffee/Americano
- Frappe
- Espresso
- Macchiato
- Any other (Pls specify)

In preparing your **Coffee**, how much did you use?

Please select one only.

- Number of spoons: 2
- Don't know
- Any other (Pls specify)

Please select the type of spoon.

Please select one only.

- Tablespoon
- Teaspoon

You indicated that you had freshly prepared **Coffee** at home. Please tell me how many people consumed the drink on that occasion?

Please select one only.

- I alone had it
- 2-3 people
- More than 3 persons
- I am not aware

Type of coffee consumed, number of spoons to prepare it, and the number of people who consumed were all captured in case of freshly prepared drinks such as coffee.

Container type, size, quantity consumed and volume

You mentioned that you had Freshly prepared **Coffee** for **1st drink Mid to late morning between about 8am and 11am**, what container did you drink from?

Please select one only.

- Disposable cup (styrofoam/plastic/paper)
- Mug/Cup/Glass
- Glass Bottle
- Plastic Bottle
- Any other

How big was the **container** that you drank from? Please select the size of the container you had your drink from.
The images are an indication of the volume of container and not the type of container.

Please select the size of the container from list.

- Small (4oz)
- Short (8oz)
- Tall (12oz)
- Grand (16oz)
- Venti (20oz)
- Any other

How much did you drink from the beverage **container**?

Please select from the options below

- Less than 1/4th of the container
- Between 1/4th and 1/2 of the container
- Between 1/2 and 3/4 th of the container
- Between 3 quarters and the full container
- Full container

SECTION 3: 12-month beverage consumption frequency questionnaire

Past year beverage consumption

Which of these other beverage types have you consumed during the past year?

Please select ALL that apply.

- [ ] Carbonated Soft Drinks caffeinated **(Cola, Non Cola, Root beer)**
- [ ] Carbonated Soft Drinks decaffeinated/caffeine free **(Cola, Non Cola, Root beer)**
- [ ] Diet Carbonated Soft Drinks decaffeinated/caffeine free **(Cola, Non Cola, Root beer)**
- [ ] Packaged Sparkling Water **(Plain Sparkling Water, Enhanced Fortified Water (eg. Glaceau), Flavored Water - regular sweetened, Flavored Water - diet sweetened with low or no calorie sweetener**
- [ ] Packaged Still Water **(Plain Bottled Water, Enhanced Fortified Water (eg. Glaceau),Flavored Water - regular sweetened, Flavored Water - diet sweetened with low or no calorie sweetener**
- [ ] Plant Water
- [ ] Still Juices and Fruit Flavored Drinks **(100% juice (single juice or vegetable),100% juice blend ,fruit flavored drinks, dairy based smoothie**
- [ ] Tea- Packaged Caffeinated **(Sweetened Regular,Sweetened Diet,Unsweetened)**
- [ ] Tea- Packaged Decaffeinated **(Sweetened Regular,Sweetened Diet,Unsweetened)**
- [ ] Prepared Tea (as available in restaurants)
- [ ] Tea- Freshly Prepared Caffeinated **(Brewed, made from powder, diet made from powder)**
- [ ] Tea- Freshly Prepared Decaffeinated/caffeine free/herbal **(Brewed, made from powder, diet made from powder)**
- [ ] Coffee- Packaged Caffeinated (Without dairy,brewed)
- [ ] Coffee- Packaged Decaffeinated (Brewed)
- [ ] Coffee- Freshly Prepared Caffeinated **(Instant powder, Brewed)**
- [ ] Coffee- Freshly Prepared Decaffeinated **(Instant powder, Brewed)**
- [ ] Plain milk consumed as a beverage
- [ ] Dairy Based Drinks **(Chocolate drinks, Packaged, Flavored milk, Packaged, Coffee drinks, Packaged (with Dairy), Yogurt Drinks, Smoothie)**
- [ ] Sports Drinks **(Regular, Diet)**
- [ ] Energy Drinks **(Regular, Diet)**
- [ ] Nutritional Beverages **(Beverage, Powder)**
- [ ] Plant Based Drinks (eg. Thai,Almond Breeze,Pacific Foods)
- [ ] None of the above

A list of all beverage types and subtypes was shown to the participant, and they were asked to select beverages consumed in the past year.

On average, in the past year, how frequently do you drink these beverages?

Please select one answer per row.

	More than once a day	Once a day	4-6 times a week	2-3 times a week	Once a week	Once in 15 days	Once a month	Less than once a month	Once a year	Never had
**Carbonated Soft Drinks caffeinated**										
Cola	○	●	○	○	○	○	○	○	○	○
Non Cola	○	○	●	○	○	○	○	○	○	○
Root Beer	○	○	○	○	○	○	○	○	○	○
**Diet Carbonated Soft Drinks caffeinated**										
Cola	○	○	●	○	○	○	○	○	○	
Non Cola	○	○	○	○	○	○	○	○	○	○
Root Beer	○	●	○	○	○	○	○	○	○	○
**Carbonated Soft Drinks decaffeinated/caffeine free**										
Cola	○	○	○	○	●	○	○	○	○	○
Non Cola	○	●	○	○	○	○	○	○	○	○
Root Beer	○	○	○	○	○	○	○	●	○	○

Closing Page

Thanks for completing the survey. This page is shown for testing only. Please close the window to exit.
